# Similarity study on lateral impact model of CFST column under high temperature

**DOI:** 10.1038/s41598-026-46522-w

**Published:** 2026-04-10

**Authors:** Junjie Wang, Jianchun Xiao, Cong Liu, Yi Yang

**Affiliations:** 1 Research Center of Space Structures, Guizhou University, Guiyang, 550025 China; 2 Guizhou Province Key Laboratory of Green Building and Intelligent Construction, Guiyang, 550025 China

**Keywords:** CFST column, Fire, Impact, Scaling, Similarity, Strain rate effect, Engineering, Materials science, Mathematics and computing

## Abstract

This paper develops a finite element model of circular concrete-filled steel tube (CFST) columns subjected to combined high temperature and lateral impact using ABAQUS through sequential thermal-stress coupling analysis, static analysis, and explicit dynamic methods, and validates it with existing experimental data from fire resistance tests and impact tests. Subsequently, based on the similarity theory of temperature fields, the heating curves for the reduced-scale circular CFST column models were designed. Meanwhile, using the dimensional system consisting of impact velocity, dynamic stress, and impact mass (*V*–*σ*_d_–*G*), an impact similarity criterion was established, and the scaling factors for key physical quantities that are closely related to the impact response were derived. After that, numerical simulations of lateral impact on the reduced-scale models were conducted at different temperatures to design reduced-scale models whose thermal–mechanical coupling response characteristics closely approximate those of the prototype structure under strain rates below the transition strain rate. Furthermore, based on the simulation results, the response differences between the reduced-scale models and the prototype, as well as the reasons for these differences, were analyzed in detail. Finally, the velocity scaling factor was derived using the strain rate-dependent constitutive equations of steel and concrete to modify the reduced-scale models, which effectively reduced the error of the reduced-scale model in predicting the prototype response.

## Introduction

Current research provides a comprehensive understanding of the performance of CFST columns under singular catastrophic events. Lie et al. ^1–3^ conducted fire resistance tests on 44 CFST columns with varying parameters, examining the effects of calculation length, material strength, load strength, eccentricity, and section type on the fire resistance of CFST columns. He also proposed a mathematical model for calculating temperature, deformation, and fire resistance of CFST columns. Han et al. ^4,5^ performed fire tests and finite element analysis on 12 CFST columns, both with and without fire protection layers, and developed calculation formulas for the fire resistance of CFST columns and fire protection layers.

Regarding the impact resistance of CFST columns, various researchers have extensively investigated the lateral impact resistance of CFST columns^6–8^, focusing on characteristics such as section geometry, material type and strength, axial load, impact load, and boundary conditions.

The combined effect of fire and impact on the mechanical properties of CFST column is more significant than their individual effects. In the study of structural mechanical behavior under the combined action of fire and impact, existing research has primarily focused on the mechanical properties and damage assessment of CFST column after cooling from high temperatures^9^. As for the impact performance of CFST column at high temperatures, current studies are mainly concentrated on axial impact condition^10–12^, with specimen sizes generally being small^13^, in contrast, experimental research on lateral impact condition at high temperatures is considerably scarce, with most relevant findings relying on finite element simulations^14^,^15^. Limited by testing conditions and costs, and enabling the small-scale specimens to more accurately reflect the mechanical response of the prototype, similarity theory has become an important approach for investigating member performance under the coupled effects of fire and impact.

Although research on the fire resistance of scaled CFST columns is currently lacking, similarity theory has been widely applied in the study of fire performance of reinforced concrete (RC) structures and composite structures. Lv^16^ utilized the pure thermal radiation model introduced by O’Connor et al.^17–19^ to perform fire experiments on reinforced concrete beams with varying proportions, and the results show that the temperature fields of the reduced-scale model and the prototype are in good agreement. Zhang et al.^20,21^ performed fire resistance test and numerical simulations on RC columns with two or three sides subjected to fire in varying quantities, and the results show that the fire resistance of the reduced-scale model and the prototype achieved good similarity. Cai et al.^22^ examined the fire resistance similarity of partially encased concrete columns across various geometric dimensions and provided a time scale adjustment equation.

In the study of the impact performance of reduced-scale models, Jones^23^ posited that the structural response of the reduced-scale model under dynamic loading deviated from conventional similarity ratios when the material exhibited strain rate sensitivity or fracture during the drop hammer impact test. To overcome the influence of strain rate sensitivity on reduced-scale models, Oshiro et al.^24^ derived the similarity relationships among various physical quantities in impact models based on the dimensional system consisting of velocity, dynamic stress, and impact mass (*V*–*σ*_d_–*G*). Zheng et al.^25^ established reduced-scale models of CFST columns under lateral impact with scaling ratios of 1/2, 1/4, and 1/8, and the results showed that the impact performance of the reduced-scale models and the prototype at room temperature achieved good similarity.

This study overcomes the limitations of a single similarity theory by integrating temperature field similarity with impact similarity, establishing for the first time an analytical model for CFST columns under the coupled effects of both factors, and verifying the reliability of similitude theory in this field. Furthermore, based on the strain rate-dependent constitutive equations of steel and concrete, the impact velocity of the reduced-scale model was modified to enable the reduced-scale model to better predict the impact performance of the prototype at high temperatures. This work aims to provide a theoretical basis for scaled model test.

## Development and validation of a high-temperature lateral impact model

### Thermal parameters and material properties

#### Steel

**Fig. 1:**
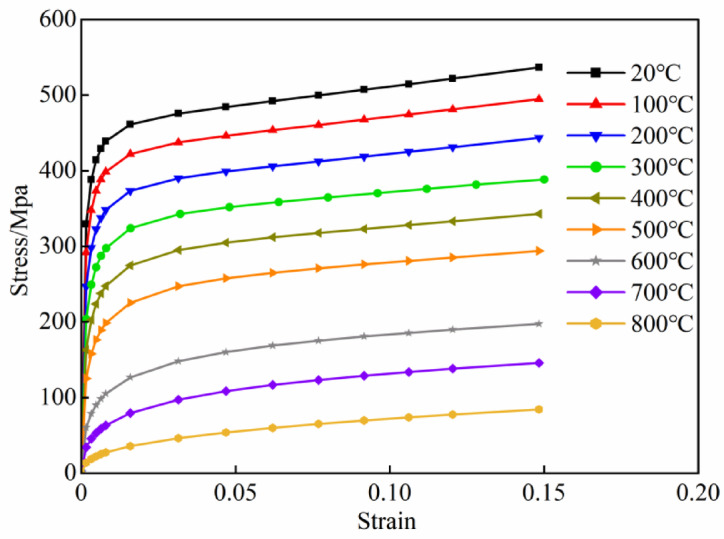
Stress–strain curves of steel.

The mechanical characteristics of steel progressively deteriorate at high temperatures. The elastic modulus of steel is derived from the reduction coefficient specified in the European standard EC2^26^. The stress–strain relationship of steel at high temperatures is articulated by the model put forth by Lie et al.^27^:

1 $$\sigma_{{\mathrm{s}}} = \left\{ {\begin{array}{*{20}l} {\frac{f(T,0.001)}{{0.001}}\varepsilon_{{\mathrm{s}}} ,} \hfill & {\varepsilon_{{\mathrm{s}}} \le \varepsilon_{{\mathrm{p}}} } \hfill \\ {\frac{f(T,0.001)}{{0.001}}\varepsilon_{{\mathrm{p}}} + f\left[ {T,(\varepsilon - \varepsilon_{{\mathrm{p}}} + 0.001} \right] - f(T,0.001),} \hfill & {\varepsilon_{{\mathrm{s}}} \ge \varepsilon_{{\mathrm{p}}} } \hfill \\ \end{array} } \right.$$

where *σ*_s_ denotes the stress intensity, *ε*_s_ represents the strain intensity, *ε*_p_ = 4 × 10^–6^ *f*_y_, *f*_y_ signifies the yield strength of steel, *f* (*T*,0.001) and *f* [*T*,(*ε*_s_ − *ε*_p_ + 0.001] are temperature-dependent functions. Taking steel with a yield strength of 360 MPa as an example, its stress–strain curves at high temperatures are shown in Fig. 1. The Cowper-Symonds calculation model is employed to consider the dynamic mechanical properties of steel at high temperatures, with the specific phrase being:

2 $$\sigma_{{\mathrm{d}}} /\sigma_{{\mathrm{s}}} { = }1{ + }\left( {\frac{{\dot{\varepsilon }}}{D}} \right)^{{1{/}p}} .$$

where *σ*_d_ represents the dynamic stress of steel at the strain rate of $\dot{\varepsilon }$,*σ*_s_ denotes the static yield stress of steel, *D* and *p* are material parameters, with values at high temperatures as suggested by Chen et al.^28^, where *D* = 400 s^−1^ and *p* = 1.

#### Concrete

The concrete utilizes the damage plastic model in ABAQUS, adopting the stress–strain relationship proposed by Han^29^, its expression is given by Eq. (3).

3 $$\left\{ {\begin{array}{*{20}l} {y = 2x - x^{2} } \hfill & {x \le 1} \hfill \\ {y = \left\{ {\begin{array}{*{20}l} {1 + q(x^{0.1\xi } - 1)} \hfill & {\xi \ge 1.12} \hfill \\ {\frac{x}{{\beta (x - 1)^{2} + x}}} \hfill & {\xi { < 1}{.12}} \hfill \\ \end{array} } \right.} \hfill & {x > 1} \hfill \\ \end{array} } \right.$$

**Fig. 2:**
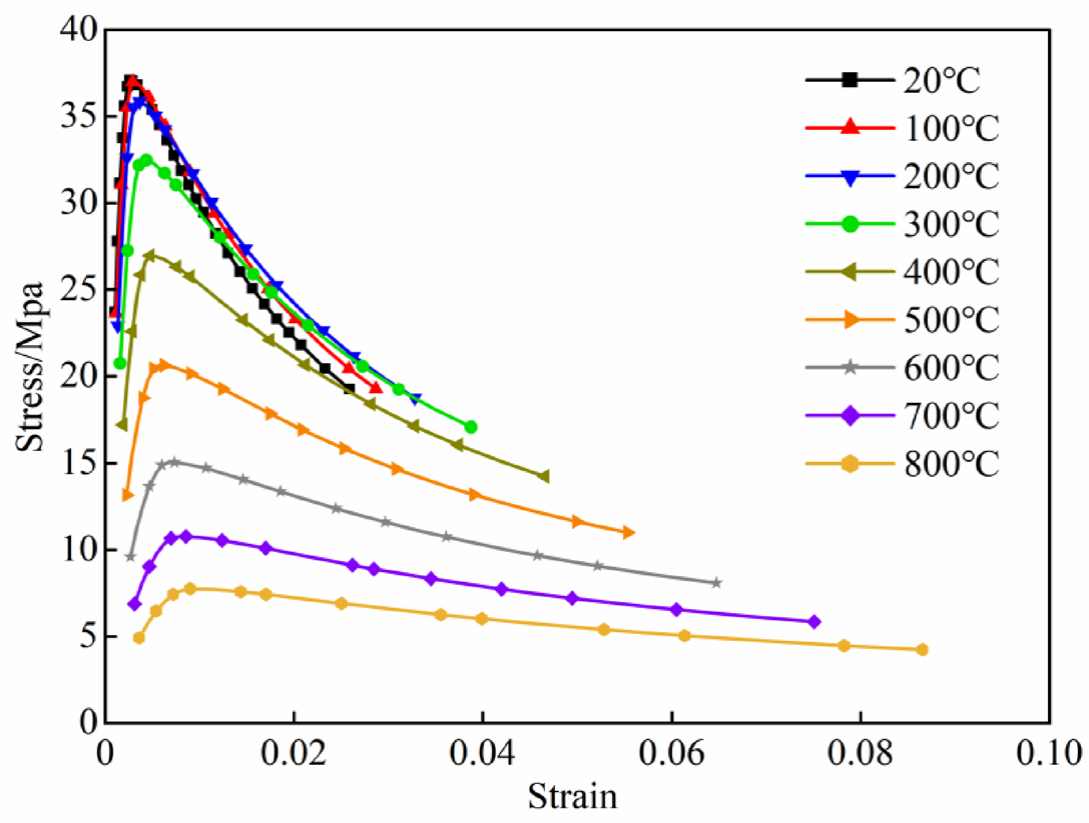
Stress–strain curves of concrete.

This relationship has been extensively used in prior research concerning the lateral impact of CFST columns at room temperature and the fire resistance. Where *x* = *ε*/*ε*_0_, *y* = *σ*/*σ*_0_, *ε*_0_ represents the ultimate strain of concrete at high temperature, *σ*_0_ represents the stress corresponding to that ultimate strain, *ξ* is the temperature-dependent confinement effect coefficient, and both *q* and *β* are parameters related to *ξ*. For their specific determination methods, see Reference^29^. Taking C45 concrete as an example, its stress–strain curves at high temperature are shown in Fig. 2.Under impact loading, the material strengthening induced by strain rate effects must be considered, the strain rate model specified in the CEB-FIB is widely adopted for room temperature conditions, as presented in Eq. (4).

4 $$\mathrm{DIF} = \frac{f_{\mathrm{cd}}}{f_{\mathrm{cs}}} = \left\{ {\begin{array}{*{20}l} {\left( {\frac{{\dot{\varepsilon }_{{\mathrm{d}}} }}{{\dot{\varepsilon }_{{{\mathrm{c0}}}} }}} \right)^{{1.026\alpha_{{\mathrm{s}}} }} ,} \hfill & {\dot{\varepsilon }_{{\mathrm{d}}} \le 30{\mathrm{s}}^{ - 1} } \hfill \\ {\gamma_{{\mathrm{s}}} \left( {\frac{{\dot{\varepsilon }_{{\mathrm{d}}} }}{{\dot{\varepsilon }_{{{\mathrm{c0}}}} }}} \right)^{1/3} ,} \hfill & {\dot{\varepsilon }_{{\mathrm{d}}} > 30{\mathrm{s}}^{ - 1} } \hfill \\ \end{array} } \right.$$

where *f*_cd_ and *f*_cs_ are the dynamic and static compressive strength of concrete, respectively. $\dot{\varepsilon }_{{\mathrm{d}}}$ is the strain rate under dynamic loading, $\alpha_{{\mathrm{s}}} = 1{/}\left( {5 + 9{f_{\mathrm{cs}}} {/}{f_{\mathrm{c0}}} } \right)$,$\dot{\varepsilon }_{{{\mathrm{c0}}}}^{(T)} = 30 \times 10^{ - 6}$. The dynamic mechanical properties of concrete at high temperatures are considered using the strength factor DIF introduced by Chen et al.^30,31^. The strength factor simultaneously accounts for the high-temperature degradation and strain rate enhancement of concrete materials. The precise expression is:

5 $$\mathrm{DIF}_T = \frac{f_{\mathrm{cd}}^T}{f_{\mathrm{cs}}^T} = \left\{ {\begin{array}{*{20}l} {\left( {\frac{{\dot{\varepsilon }_{{\mathrm{c}}}^{T} }}{{\dot{\varepsilon }_{{{\mathrm{c0}}}}^{T} }}} \right)^{{1.026a_{{\mathrm{s}}} }} ,} \hfill & {\dot{\varepsilon }_{{\mathrm{c}}}^{T} \le \dot{\varepsilon }_{{\text{trans }}}^{T} \;\left( {20^{^\circ } {\mathrm{C}} \le T \le 800^{^\circ } {\mathrm{C}}} \right)} \hfill \\ {\gamma_{{\mathrm{s}}} \left( {\frac{{\dot{\varepsilon }_{{\mathrm{c}}}^{T} - g\left( T \right)}}{{\dot{\varepsilon }_{{{\mathrm{c0}}}}^{T} }}} \right)^{h\left( T \right)} ,} \hfill & {\dot{\varepsilon }_{{\mathrm{c}}}^{T} > \dot{\varepsilon }_{{\text{trans }}}^{T} \;\left( {20^{^\circ } {\mathrm{C}} \le T \le 800^{^\circ } {\mathrm{C}}} \right)} \hfill \\ \end{array} } \right.$$

where $\dot{\varepsilon }^{(T)}_{{\mathrm{c}}}$ is the strain rate at temperature *T*; ${\mathrm{lg}}\gamma_{s} = 6.156\alpha_{{\mathrm{s}}} - 2$,$\alpha_{{\mathrm{s}}} = 1{/}\left( {5 + 9f_\mathrm{cs}^{{{T}}} {/}f_{cT0} } \right)$, $f_\mathrm{cs}^{{{T}}}$ is the quasi-static strength at temperature *T*, *f*_*cT*0_ = 10 MPa, $\dot{\varepsilon }_{{{\mathrm{c0}}}}^{(T)} = 30 \times 10^{ - 6}$; $\dot{\varepsilon }^{(T)}_{{{\mathrm{trans}}}}$ is the conversion strain rate at time *T*; *g*(*T*) and *h*(*T*) are temperature-dependent equations and can be found in^30^. Furthermore, the thermal parameters for steel and concrete, including thermal conductivity, specific heat, thermal expansion coefficient, and density, were adopted from Reference^29^, as detailed in Table 1.

**Table 1:** Thermal parameters used in the fire and the impact model.

Thermal parameters	Steel	Concrete
Thermal conductivity	$$\lambda_{{\mathrm{s}}} =$$$$\left\{ {\begin{array}{*{20}l} {48 - 0.0022T} \hfill & {0 \le T \le 900^{ \circ } {\mathrm{C}}} \hfill \\ {28.2} \hfill & {T \ge 900^{ \circ } {\mathrm{C}}} \hfill \\ \end{array} } \right.$$	$$\lambda_{{\mathrm{c}}} =$$$$\left\{ {\begin{array}{*{20}l} {1.355} \hfill & {0^{ \circ } {\mathrm{C}} \le T \le 293^{ \circ } {\mathrm{C}}} \hfill \\ { - 0.001241T + 1.7162} \hfill & {T > 293^{ \circ } {\mathrm{C}}} \hfill \\ \end{array} } \right.$$
Specific heat	$$\rho_{{\mathrm{s}}} c_{{\mathrm{s}}} =$$$$\left\{ {\begin{array}{*{20}l} {(0.004T + 3.3) \times 10^{6} \;{\mathrm{J/(m}}^{3} \;^{ \circ } {\mathrm{C)}}} \hfill & {{0}^{ \circ } {\mathrm{C}} \le T \le 650^{ \circ } {\mathrm{C}}} \hfill \\ {(0.068T - 38.3) \times 10^{6} \;{\mathrm{J/(m}}^{3} \;^{ \circ } {\mathrm{C)}}} \hfill & {{650}^{ \circ } {\mathrm{C}} \le T \le 725^{ \circ } {\mathrm{C}}} \hfill \\ {( - 0.086T + 73.35) \times 10^{6} \;{\mathrm{J/(m}}^{3} \;^{ \circ } {\mathrm{C)}}} \hfill & {{725}^{ \circ } {\mathrm{C}} \le T \le 800^{ \circ } {\mathrm{C}}} \hfill \\ {4.55 \times 10^{6} \;{\mathrm{J/(m}}^{3} \;^{ \circ } {\mathrm{C)}}} \hfill & {T{ > }800^{ \circ } {\mathrm{C}}} \hfill \\ \end{array} } \right.$$	$$\rho_{{\mathrm{c}}} c_{{\mathrm{c}}} =$$$$\left\{ {\begin{array}{*{20}l} {2.566 \times 10^{6} \;{\mathrm{J/(m}}^{3} \,^{ \circ } {\mathrm{C)}}} \hfill & {{0}^{ \circ } {\mathrm{C}} \le T \le 400^{ \circ } {\mathrm{C}}} \hfill \\ {(0.1765T - 68.034) \times 10^{6} \;{\mathrm{J/(m}}^{3} \,^{ \circ } {\mathrm{C)}}} \hfill & {{400}^{ \circ } {\mathrm{C}} \le T \le 410^{ \circ } {\mathrm{C}}} \hfill \\ {( - 0.05043T + 25.00671) \times 10^{6} \;{\mathrm{J/(m}}^{3} \,^{ \circ } {\mathrm{C)}}} \hfill & {{410}^{ \circ } {\mathrm{C}} \le T \le 445^{ \circ } {\mathrm{C}}} \hfill \\ {2.566 \times 10^{6} \;{\mathrm{/(m}}^{3} \,^{ \circ } {\mathrm{C)}}} \hfill & {{445}^{ \circ } {\mathrm{C}} \le T \le 500^{ \circ } {\mathrm{C}}} \hfill \\ {(0.01603T - 5.44881) \times 10^{6} \;{\mathrm{J/(m}}^{3} \,^{ \circ } {\mathrm{C)}}} \hfill & {{500}^{ \circ } {\mathrm{C}} \le T \le 635^{ \circ } {\mathrm{C}}{\kern 1pt} } \hfill \\ {(0.16635T - 100.90225) \times 10^{6} \;{\mathrm{J/(m}}^{3} \,^{ \circ } {\mathrm{C)}}} \hfill & {{635}^{ \circ } {\mathrm{C}} \le T \le 715^{ \circ } {\mathrm{C}}} \hfill \\ {( - 0.22103T + 176.07343) \times 10^{6} \;{\mathrm{J/(m}}^{3} \,^{ \circ } {\mathrm{C)}}{\kern 1pt} } \hfill & {{715}^{ \circ } {\mathrm{C}} \le T \le 785^{ \circ } {\mathrm{C}}} \hfill \\ {2.566 \times 10^{6} \;{\kern 1pt} {\mathrm{J/(m}}^{3} \,^{ \circ } {\mathrm{C)}}} \hfill & {T{ > }785^{ \circ } {\mathrm{C}}} \hfill \\ \end{array} } \right.$$
Expansion coefficient	$$\alpha_{{\mathrm{s}}} = \left\{ {\begin{array}{*{20}l} {(0.004T + 12) \times 10^{ - 6} \;{\mathrm{m/}}({\mathrm{m}}\;^{ \circ } {\mathrm{C}})} \hfill & {T{ < 1000}^{ \circ } {\mathrm{C}}} \hfill \\ {16 \times 10^{ - 6} \;{\mathrm{m/}}({\mathrm{m}}\;^{ \circ } {\mathrm{C}}){\kern 1pt} } \hfill & {T \ge {1000}^{ \circ } {\mathrm{C}}} \hfill \\ \end{array} } \right.$$	$\alpha_{{\mathrm{c}}} = (0.008T + 6) \times 10^{ - 6} \;{\mathrm{m/}}({\mathrm{m}} \cdot^{ \circ } {\mathrm{C}})$
Density	$\rho_{{\mathrm{c}}} = 7850{\kern 1pt} {\kern 1pt} {\kern 1pt} {\mathrm{kg}}{\kern 1pt} {\mathrm{/m}}^{3}$	$\rho_{{\mathrm{c}}} = 2400{\kern 1pt} {\kern 1pt} {\kern 1pt} {\mathrm{kg}}{\kern 1pt} {\mathrm{/m}}^{3}$

Compared to steel, the influence of strain rate on concrete at high temperatures is more complex. This is primarily attributed to the thermal inertia of concrete material. When heated, the surface temperature of concrete members rises rapidly, while heat transfer to the interior proceeds relatively slowly. Consequently, an uneven temperature field develops within the concrete members. This makes it particularly challenging to select a representative temperature value of the concrete cross-section for calculating the dynamic amplification factor and for subsequent strain rate corrections. Chen et al.^30^ research indicates that the strain rate of concrete gradually increases with rising temperature, and at room temperature, the strain rate of concrete is approximately 30 s^−1^.When the strain is below the transition strain rate, the DIF curves (Eq. 5) at various high temperatures almost coincide with the curve suggested by the CEB formula (Eq. 4) for room temperature. Consequently, the investigation focuses solely on the similarity of the dynamic response between the prototype and the reduced-scale models of the CFST column when the actual strain rate is below the transition strain rate (low strain rate). The examples presented in Chapter 3 are derived via meticulously selected analysis to ascertain the low strain rate response of CFST column under low-speed impact, ensuring that the column transitions into the plastic stage without experiencing instability failure.

### Establishment of FE model

**Fig. 3:**
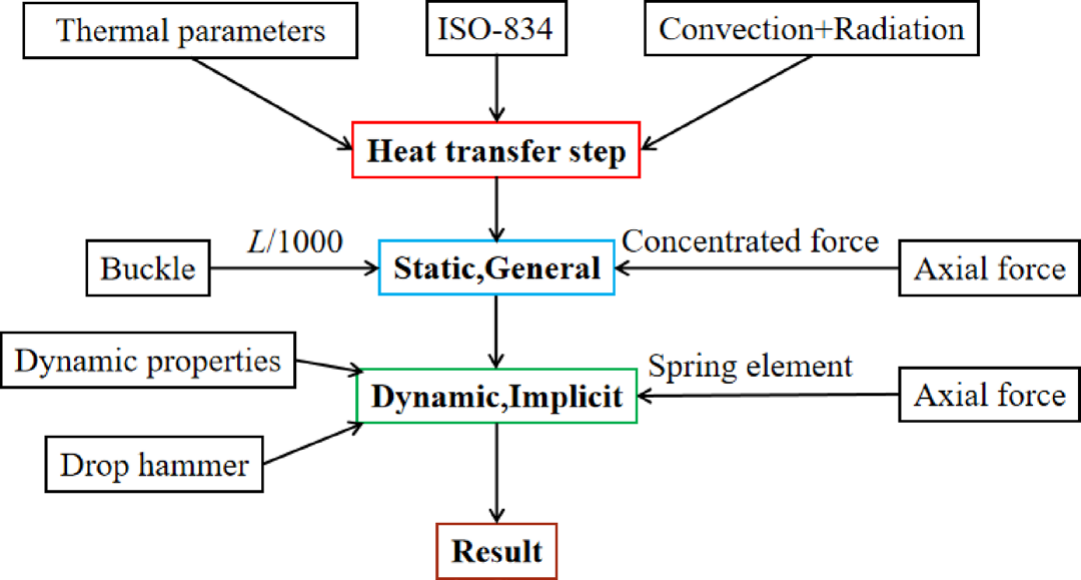
Process of coupled high temperature and impact.

**Fig. 4:**
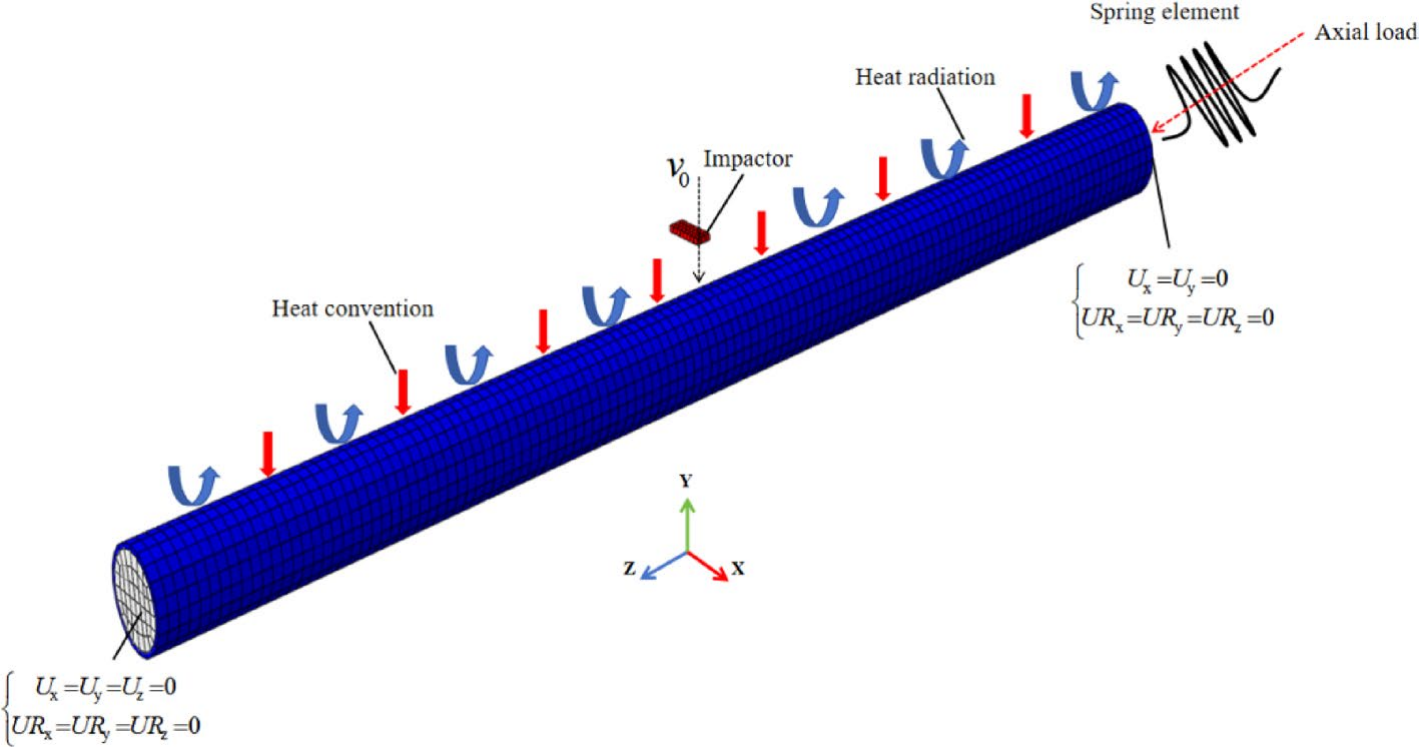
Lateral impact model of CFST column under fire condition.

The static analysis module of ABAQUS/Standard enables the execution of fire resistance analysis for members through sequential thermal–mechanical coupling or comprehensive thermal–mechanical coupling. Zhang^32^ indicates that the two methods both use the “tie” constraint to realize the temperature transfer between the steel tube and the concrete surface without considering the contact thermal resistance, and the temperature field results of the two are not much different. However, this contact relationship will lead to significantly discrepancies in fire resistance (Espinos et al.^33^), as the steel tube and concrete experience relative sliding under the influence of axial force and high temperatures. To achieve the transition of contact methods, this paper will adopt a sequential thermal–mechanical coupling approach to establish the fire model under axial loading, then the results will be imported into the lateral impact model to simulate the impact process. Figure 3 illustrates the coupling process, whereas Fig. 4 depicts the FE model.

The fire model employs the “Heat transfer” step and the ISO-834 standard heating curve to simulate fire environment, the heat transfer mode involves both convective and radiative heat transfer. The comprehensive radiation coefficient is 0.7, the convection coefficient is 25 W/m^2^, the surfaces of the steel tube and concrete are set as tied, and the temperature field of the component is calculated. The initial defects of the members were considered by importing the predefined fields with the initial buckling of *L*/1000^34^ by using the “Buckle” analysis step, and the axial force at the end was applied by the spring element^35^. The temperature field of the members was imported as the predefined field into the model with the “Static, General” as the analysis step, the hard contact interaction is defined between the steel tube and concrete, with a friction coefficient of 0.6^33^, the fire resistance limit of the component is then calculated.

In the impact model, the “Dynamic, Explicit” step is selected, the calculation results (*restart) file from the fire model are imported into the impact model as “the initial state” using the (*input) command. The model’s dynamic material properties are modified, and the axial force is provided via the spring element to prevent axial force unloading during the impact process, and the spring displacement should be reset to a value equal to the displacement at the previous step, reduced by the axial deformation of the component. Gravity is applied to the entire model; the drop hammer was positioned 2 mm above the mid-span of the member and was initialized with a downward velocity. The contact interfaces between the drop hammer and the steel tube are set as “general contact” interaction, for the contact interface between the outer surface of the steel tube and the impactor, a hard contact condition with no friction is defined, for the interface between the steel tube and the concrete, hard contact is applied with a friction coefficient of 0.6. The principal parameters of the model are presented in Table 2.

**Table 2:** Key settings in the FE model.

Models	Analyze steps	Interactions	Element types			Boundary condition
			Steel tube	Concrete	Hammer	
Fire model	Heat transfer	“Tie” constraint	DS4	DC3D8		Fixed at both ends
	Static, General	Surface to surface	S4R	C3D8R		
Impact model	Dynamic, Explicit	General contact	S4R	C3D8R	C3D8R	Fixed at both ends

Currently, there is no actual experimental research on high-temperature lateral impact in this field. Therefore, this paper will conduct separate simulation validations: one for existing fire tests on CFST columns, and the other for lateral impact tests performed at room temperature.

### Verification of the temperature field and fire resistance model

**Fig. 5:**
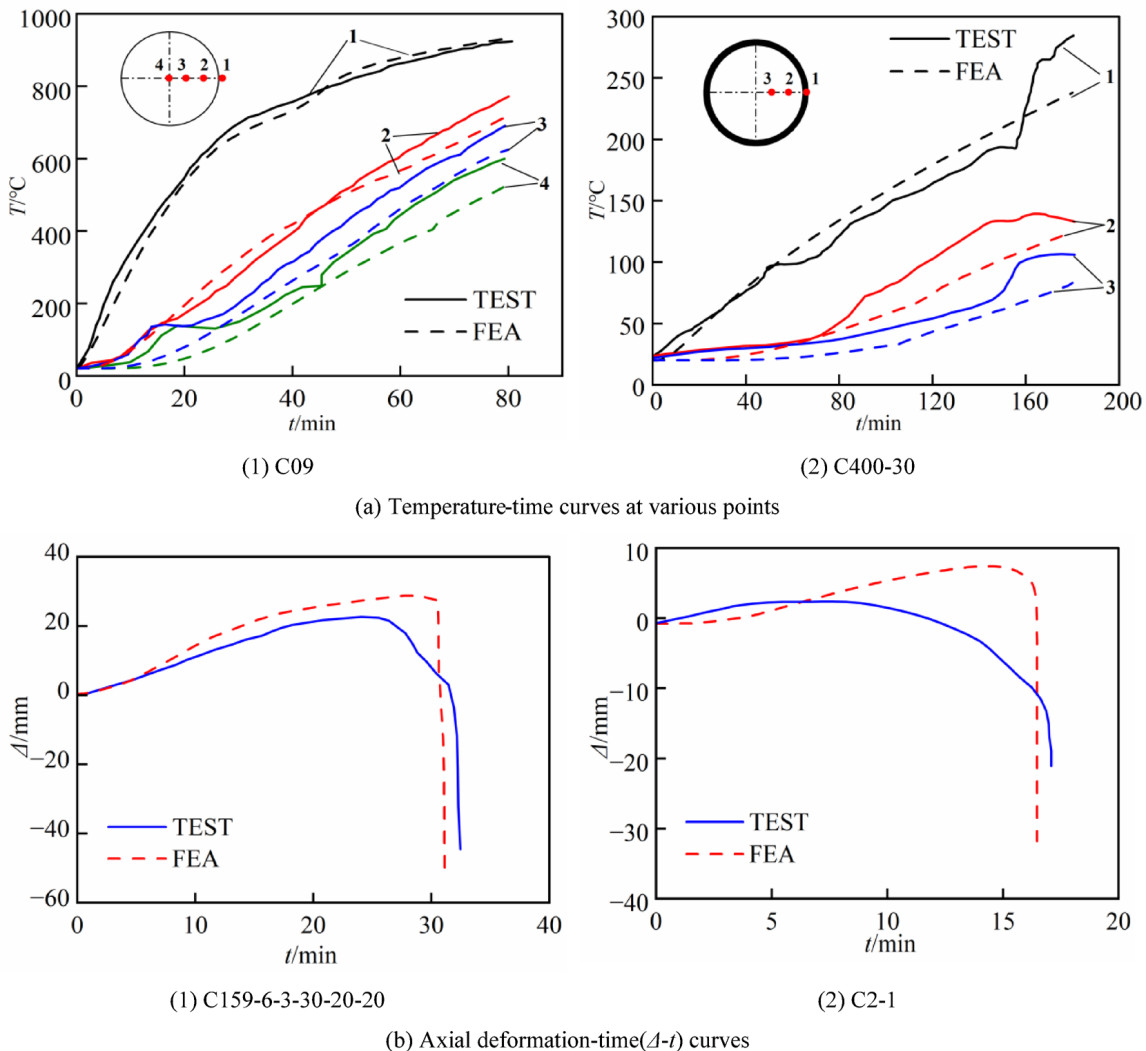
Comparison between predicted and test curves.

The fire temperature field from ^2,4^ and the fire resistance from^36,37^ are chosen for validation. The outer surface of the steel tube of the specimen is subjected to heating in accordance with the ISO-834 standard heating curve. Table 3 presents the specimens information from the experiment, where *D* denotes the outer diameter of the component, *t*_s_ represents the thickness of the steel tube, *L* indicates the length of the specimens, *N* signifies the axial force,* e* is load eccentricity, *a* refers to the thickness of the protective layer, *t*_Exp_ and *t*_FE_ represent the measured value of fire resistance and the simulated value of fire resistance. Figure 5a illustrates the comparison between the measured temperature values (solid line) and the simulated values (dashed line) at various measuring points of the specimens, and Fig. 5b depicts the comparison between the experimental and simulated results of the axial displacement–time curve of the specimens. Figure 5a illustrates that the simulated temperature profiles for each measurement point of the two specimens align with the trends of the measured temperature curves from the test, demonstrating that this method effectively simulates the temperature field of concrete-filled steel tubes at high temperatures. As can be seen from Fig. 5b, the axial deformation-time curve obtained from the finite element simulation agrees with the experimental curve in overall trend, the simulated fire resistance time is less than the experimentally measured value, with differences of 0.8 min and 0.3 min, respectively. The simulated value of axial deformation is greater than the measured value, with a discrepancy not exceeding 10 mm, and the deviation is negligible for a member with a length of 3810 mm. Additionally, it can be seen that the axial deformation is in the opposite direction to the axial force, indicating that it primarily arises from thermal expansion of the material rather than from plastic deformation induced by axial loading. This deformation remains within the elastic range and does not significantly influence the constitutive behavior—such as the strength or elastic modulus—of either the steel or the concrete. The reason is that the supports of the test were not ideal “pin supports” due to installation, aging and other factors, the rotational restraint stiffness was not zero, which implies that the constraint conditions in the test were stronger than those in the finite element model. Overall, the simulated fire resistance time agrees well with the experimentally measured value, demonstrating that the fire resistance model has good accuracy.

**Table 3:** Information and results of the fire tests.

Specimen	*D* × *t*_s_ (mm)	*f*_y_ (MPa)	*f*_cu_ (MPa)	*L* (mm)	*N* (kN)	*a* (mm)	*e* (mm)	*t*_Exp_ (min)	*t*_FE_ (min)	Reference
C09	186.3 × 6.35	350	28.6	3810		0				Lie^2^
C400-30	400 × 6	293	41.3	3810		30				Han^4^
C159-6–3-30–20-20	159 × 6	275	38.25	3810	252.8	0	50	32	31.2	Moliner^37^
C2-1	219 × 5	290.3	41.3	3810	450	0	32.85	17	17.3	Han^36^

### Validation of the side impact model

**Fig. 6:**
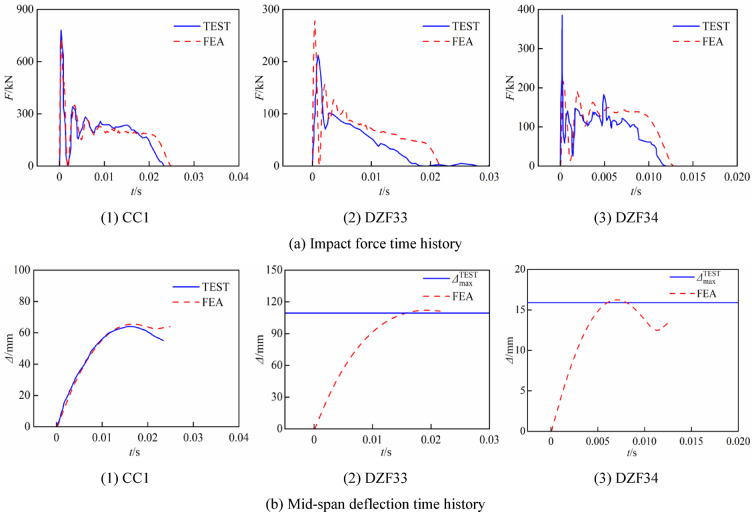
Comparison between predicted and test time history curves.

**Fig. 7:**
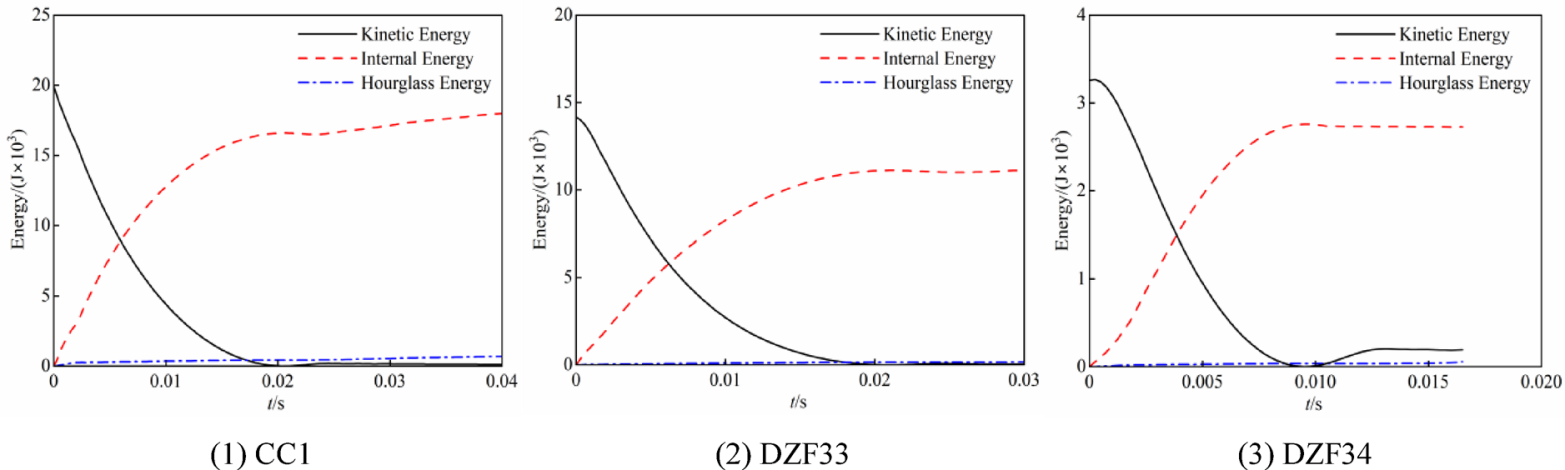
Energy curves for the specimens in the finite element simulation.

The verification of the finite element simulation was conducted by selecting the lateral impact test of CFST columns at room temperature from sources^6^ and^7^. The component parameters, test data and simulation results are shown in Table 4, where *D* is the outer diameter of the component, *t*_s_ is the thickness of the steel tube, *N* is the axial force, *h* and *M* are the release height and mass of the drop hammer respectively, *F*_p_ is the platform value of the impact force curve, *Δ*_max_ is the maximum deflection at the mid-span of the specimens after being impacted, and *t* is the impact time. Figure 6 illustrates the comparative results of the impact force and mid-span deflection time history curves for several specimens. The simulation curves trend is largely congruent with that of the experimental curves. The significant axial force results in the absence of a distinct platform section in the impact time history curve of DZF33, and the simulated curve also verifies this well. The mean ratios of the test value to the simulation value for the impact force platform, the peak mid-span deflection, and the impact time are 1.021, 0.974, and 0.950, respectively. The energy time-history curves for each specimen are shown in Fig. 7, with the ratio of hourglass energy to internal energy remaining below 5% in all cases. In general, the finite element model effectively simulates the lateral impact process of concrete-filled steel tube members.

**Table 4:** Information and results of the lateral impact tests.

Specimen	*D* × *t*_s_ (mm)	*f*_y_ (MPa)	*f*_cu_ (MPa)	*N* (kN)	*h* (m)	*M* (kg)	*F*_p_ (kN)		*Δ*_max_ (mm)		*t* (ms)		Reference
							Test	FE	Test	FE	Test	FE	
CC1	180 × 3.65	247	75.1	0	5.5	465	228.4	218.5	64	65.5	23.3	24.8	
CC2	180 × 3.65	247	75.1	0	2.5	920	229.1	212.8	70	73	34.6	35.4	Hou^7^
CC3	180 × 3.65	247	75.1	0	8	465	210.4	195.9	91	93.2	29.6	30.7	
DZF31	114 × 3.5	298	47.5	200	7	229.8	125.7	131.4	101.7	104.3	38.2	38.5	
DZF33	114 × 3.5	298	47.5	400	7	229.8	**—**	**—**	109.4	112.1	18.6	21.5	Wang^6^
DZF34	114 × 3.5	298	47.5	200	1	229.8	127.6	134.0	15.9	16.2	12.3	12.8

## Criterion for temperature field similarity

### Derivation of similarity criterion

Shape factors, as established by Baker^38^, are widely recognized and utilized across various disciplines. The shape similarity factor is defined as *β*_*l*_ = *l*_m_/*l*_p_, where* l*_m_ and *l*_p_ denote the dimensions of the reduced-scale model and the prototype, respectively, with the subscript p indicating the prototype and m indicating the reduced-scale model.

The similarity of the temperature field indicates that the temperature distribution between the reduced-scale model and the prototype is consistent, requiring that the cross-sectional temperature of the corresponding section in both the reduced-scale models and the prototype should maintain identical at any given time during fire duration time, denoted as *T*_m_ = *T*_p_. The instantaneous heat flux inside the member is determined by the rate of change of its surface temperature *T*_w_ (∂*T*_w_/∂t). Fourier’s second law indicates that the integral analogy approach can be employed to derive:

6 $$F_{o} = \frac{{\lambda \left( {\frac{{\partial^{2} T}}{{\partial x^{2} }}} \right)}}{{\rho c\frac{{\partial T_{{\mathrm{w}}} }}{\partial t}}} = \frac{\lambda }{\rho c}\frac{t}{{l^{2} }}\frac{T}{{T_{{\mathrm{w}}} }}$$

where *F*_o_ represents the Fourier number, *t* denotes time, *l* signifies the length of the item in a certain direction, *ρ* indicates density, *c* and *λ* refer to specific heat capacity and thermal conductivity, ∂^2^*T*/∂*x*^*2*^ describes the curvature of the temperature distribution within the component section. The third law of similarity states that a phenomenon is considered similar if the criterion for similarity is identical, so it can be derived:

7 $$\left( {\frac{{l_{{}}^{2} }}{{t_{{}} }} \cdot \frac{{T_{{\mathrm{w}}} }}{T}} \right)_{{\mathrm{m}}} = \left( {\frac{{l_{{}}^{2} }}{{t_{{}} }} \cdot \frac{{T_{{\mathrm{w}}} }}{T}} \right)_{{\mathrm{p}}}$$

### Temporal similarity

McGuire et al.^39^ posited that when the corresponding points of the reduced-scale model and the prototype attained identical temperatures, the fire resistance duration of the model was proportional to the square of the geometric ratio, necessitating an adjustment of the furnace temperature appropriately. O’Connor et al.^17–19^ modified the heating curve of the reduced-scale model using the thermal radiation model, the thermal convection model, and the mixed model for the RC structure, and conducted pertinent experiments. The test demonstrated that the modified heating curve could maintain the temperature distribution of the reduced-scale model in alignment with that of the prototype. In conjunction with Eq. (7), the reduced-scale model and the prototype must remain consistent temperature field distributions, indicating a corresponding relationship in their equivalent fire resistance time:

8 $$\left( {\frac{{l^{2} }}{t}} \right)_{{\mathrm{m}}} = \left( {\frac{{l^{2} }}{t}} \right)_{{\mathrm{p}}}$$

Equation (8) can be transformed into:

9 $$t_{{\mathrm{m}}} = t_{{\mathrm{p}}} \cdot \frac{{l_{{\mathrm{m}}}^{2} }}{{l_{{\mathrm{p}}}^{2} }} = t_{{\mathrm{p}}} \cdot \beta_{l}^{2}$$

Equation (9) demonstrates that the actual heating duration of the reduced-scale model is derived by scaling the heating duration of the prototype in accordance with the reduced-scale ratio.

### Methodology for calculating the heating curve of a reduced-scale model

Heat flux density *q*_n_ of thermal conduction in the specimen is:

10 $$q_{{\mathrm{n}}} = \lambda \frac{dT}{{dx}}$$

As the temperatures at corresponding points of the reduced-scale model and the prototype are identical during the heat conduction process, the temperature gradient *dT*/*dx* of the prototype is inversely proportional to that of the reduced-scale model. Similarly, the heat flux density qn exhibits the same inverse proportionality, which can be articulated by the following formula:

11 $$q_{{{\mathrm{n}},{\mathrm{m}}}} = \frac{1}{{\beta_{l} }}q_{{{\mathrm{n}},{\mathrm{p}}}}$$

At the boundary between the furnace air and the specimen, heat transmission occurs through convection and radiation, resulting in a heat flux density comprised of convective heat transfer *q*_c_ and radiative heat transfer *q*_r_:

12 $$q_{{\mathrm{n}}} = q_{{\mathrm{c}}} + q_{{\mathrm{r}}}$$

According to Newton’s law of cooling:

13 $$q_{{\mathrm{c}}} = h\left( {T_{{\mathrm{f}}} - T_{{\mathrm{w}}} } \right) \,$$

where *h* is the convective heat transfer coefficient, *T*_f_ and *T*_w_ are the furnace temperature and the surface temperature of the specimen, respectively.

According to Stefan-Boltzmann law:

14 $$q_{{\mathrm{r}}} = \sigma \varepsilon \left( {T_{{\mathrm{f}}}^{4} - T_{{\mathrm{w}}}^{4} } \right)$$

where *ε* is the radiation heat transfer coefficient of the object, *σ* is the Stefan-Boltzmann constant, valued at 5.67 × 10^–8^ W/(m^2^ K^4^). Substituting **Eqs. **(12), (13) and (14) into Eq. (11) yields:

15 $$h\left( {T_{{{\mathrm{f}},{\mathrm{m}}}} - T_{{{\mathrm{w}},{\mathrm{m}}}} } \right) + \varepsilon \sigma \left( {T_{{{\mathrm{f}},{\mathrm{m}}}}^{4} - T_{{{\mathrm{w}},{\mathrm{m}}}}^{4} } \right) = \frac{1}{{\beta_{l} }}\left( {h\left( {T_{{{\mathrm{f}},{\mathrm{p}}}} - T_{{{\mathrm{w}},{\mathrm{p}}}} } \right) + \varepsilon \sigma \left( {T_{{{\mathrm{f}},{\mathrm{p}}}}^{4} - T_{{{\mathrm{w}},{\mathrm{p}}}}^{4} } \right)} \right)$$

where *T*_f,m_ is the furnace temperature of the reduced-scale model, *T*_f,p_ is the furnace temperature of prototype, and *T*_w,p_ is the surface temperature of the prototype.

Equation (15) accounts for the combined effects of thermal convection and radiation. Since the reduced-scale models and the prototype share an identical temperature field distribution, *T*_w,m_ = *T*_w,p_. This implies that the heating curve of the reduced-scale models,* T*_f,m_, can be obtained simply by collecting the surface temperature data of the prototype and the furnace temperature. However, in practical applications, it is difficult to determine the respective contributions of thermal convection and radiation, and both the convective heat transfer coefficient *h* and the radiative heat transfer coefficient *ε* are also difficult to determine.

Lv^16^ indicates that the temperature fields of the reduced-scale models show good similarity with that of the prototype when a pure thermal radiation model is adopted, accompanied by the provision of the heat radiation formula:

16 $$T_{{{\mathrm{f}},{\mathrm{m}}}} = \sqrt[4]{{T_{{{\mathrm{w}},{\mathrm{p}}}}^{4} + \frac{1}{{\beta_{l} }}\left( {T_{{{\mathrm{f}},{\mathrm{p}}}}^{4} - T_{{{\mathrm{w}},{\mathrm{p}}}}^{4} } \right)}} \,$$

**Fig. 8:**
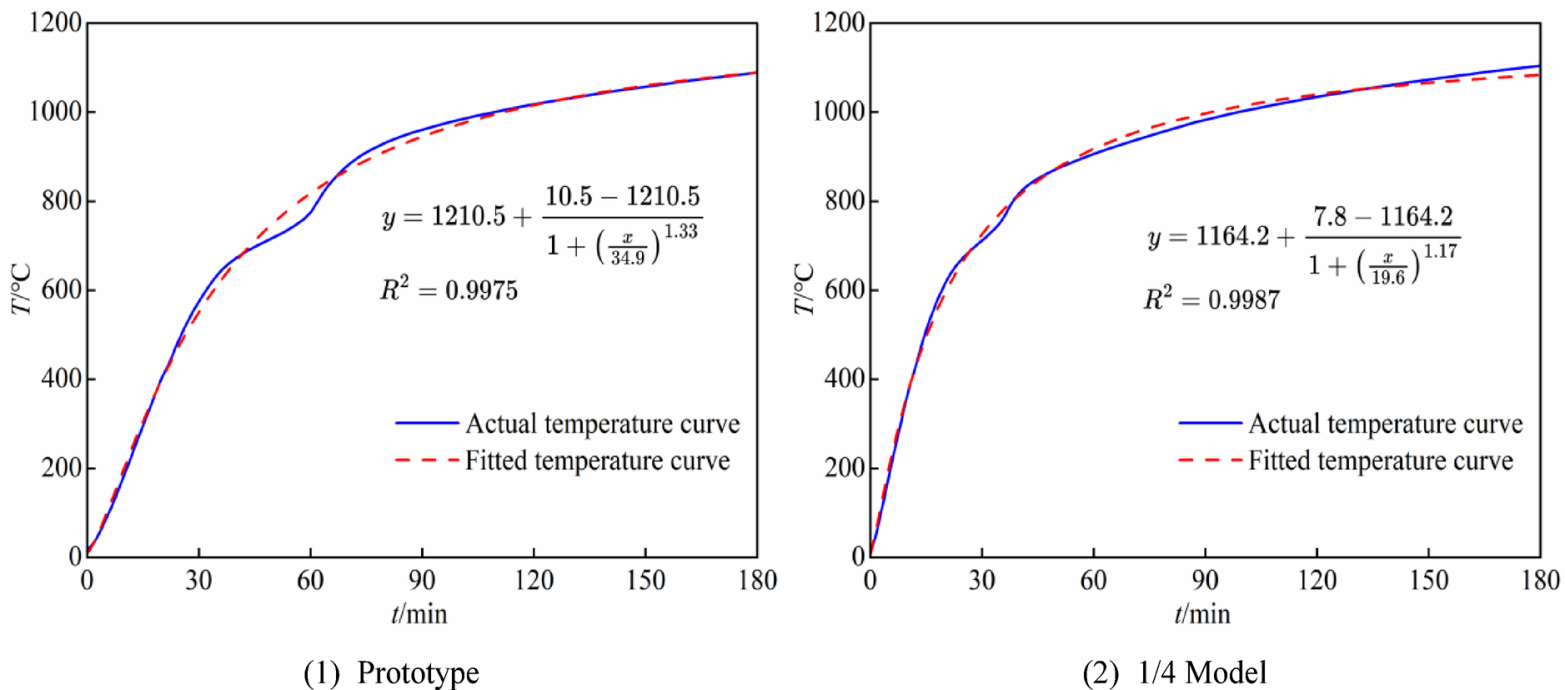
Fitting curves of the outer surface temperature of the steel tube.

From Eq. (16), it can be seen that when the prototype furnace temperature is known (using the ISO-834 standard heating curve), the heating curves of the reduced-scale models *T*_f,m_ can be obtained by collecting the temperature data of the outer surface of the prototype steel tube at corresponding time points and fitting the data using ORIGIN software. After extensive simulation and analysis, this paper ultimately selects the reduced-scale models with shape factors of 1/4, 1/3, and 1/2 to conduct similar research on the temperature field. The fitting results of the steel tube outer surface temperature are shown in Fig. 8, demonstrating good fitting performance.

**Fig. 9:**
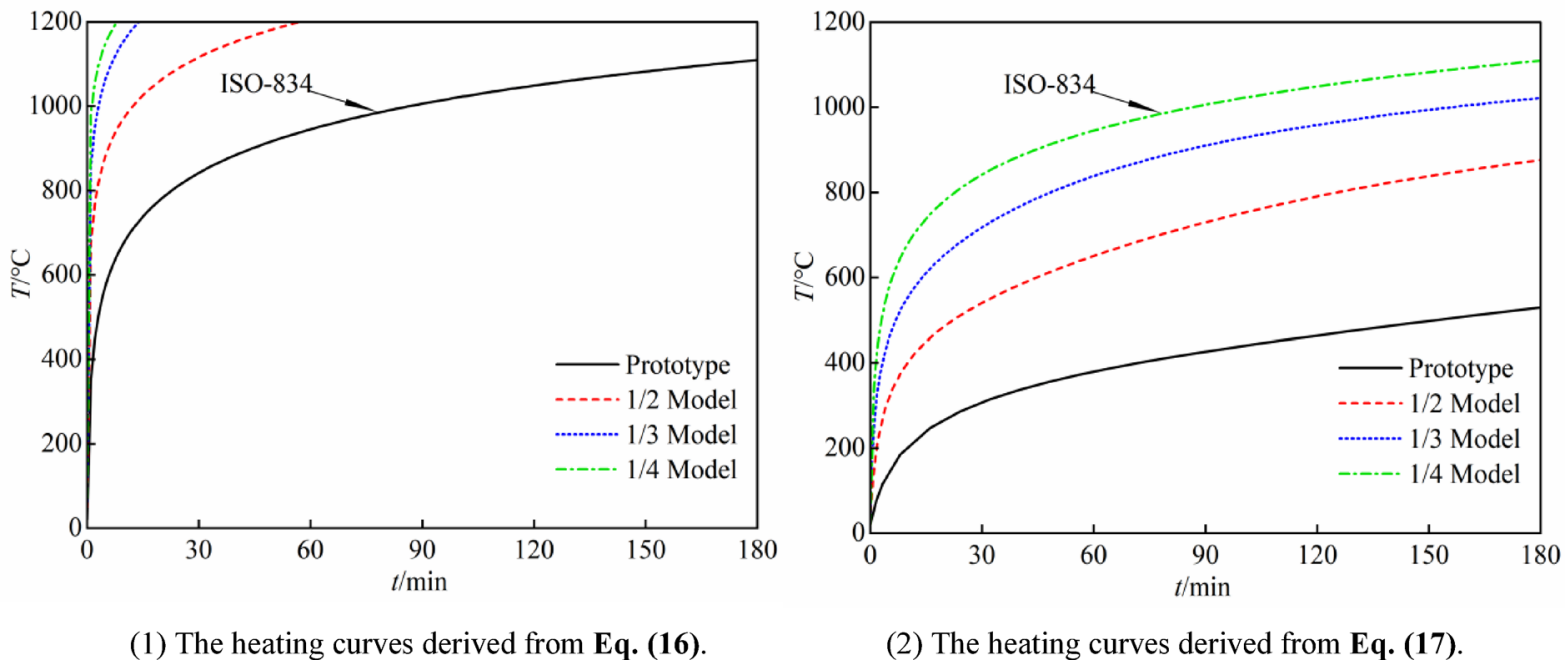
Temperature heating curves.

However, from Eqs. (16) and (9), it can be seen that after scaling the heating time for the reduced-scale model, its heating rate becomes significantly greater than that of the prototype. Furthermore, as the scaling ratio decreases further, the heating rate increases rapidly, to the extent that the heating power of the combustion furnace will be unable to meet the demand, this makes conducting experiments extremely difficult. Figure 9(1) shows the heating curve of the reduced-scale models derived from Eq. (16).

Let the shape factors of the prototype, 1/2 reduced-scale model, 1/3 reduced-scale model and 1/4 reduced-scale model be $\beta_{{l_{1} }}$,$\beta_{{l_{2} }}$,$\beta_{{l_{3} }}$ and $\beta_{{l_{4} }}$.It follows that:

$$\beta^{\prime}_{{l_{1} }} = \frac{{\beta_{{l_{4} }} }}{{\beta_{{l_{1} }} }} = \frac{1}{4},\beta^{\prime}_{{l_{2} }} = \frac{{\beta_{{l_{4} }} }}{{\beta_{{l_{2} }} }} = \frac{1}{2},\beta^{\prime}_{{l_{3} }} = \frac{{\beta_{{l_{4} }} }}{{\beta_{{l_{3} }} }} = \frac{3}{4}$$

Convert Eq. (16) into:

17 $$T_{{{\mathrm{f}},\beta^{\prime}_{l} }} = \sqrt[4]{{T_{{{\mathrm{w}},1/4}}^{4} + \beta^{\prime}_{l} \left( {T_{{{\mathrm{f}},1/4}}^{4} - T_{{{\mathrm{w}},1/4}}^{4} } \right)}}$$

where *T*_w,1/4_ represents the outer surface temperature of the steel tube of the 1/4 reduced-scale model; *T*_f,1/4_ represents the furnace temperature of the 1/4 reduced-scale model, namely, the ISO-834 standard heating curve; in $T_{{{\mathrm{f,}}\beta^{\prime}_{l} }}$, the values of $\beta^{\prime}_{l}$ are 1/4, 1/2, and 3/4, corresponding to the furnace temperatures of the prototype, the 1/2 reduced-scale model, and the 1/3 reduced-scale model, respectively.

According to Eq. (17), if the 1/4 reduced-scale model is heated using the ISO-834 standard heating curve, and the outer surface temperature data of the steel tube are collected to derive the heating curves for the prototype and other reduced-scale models, the heating rate of the resulting curves will inevitably be lower than that of the ISO-834 standard heating curve. Figure 9(2) shows the heating curves of the reduced-scale models derived from Eq. (17), it can be seen that this method can perfectly solve the problem of insufficient power output of the combustion furnace. Subsequent research in this paper will also adopt the heating curve derived by this method.

### Similarity test of fire model

**Fig. 10:**
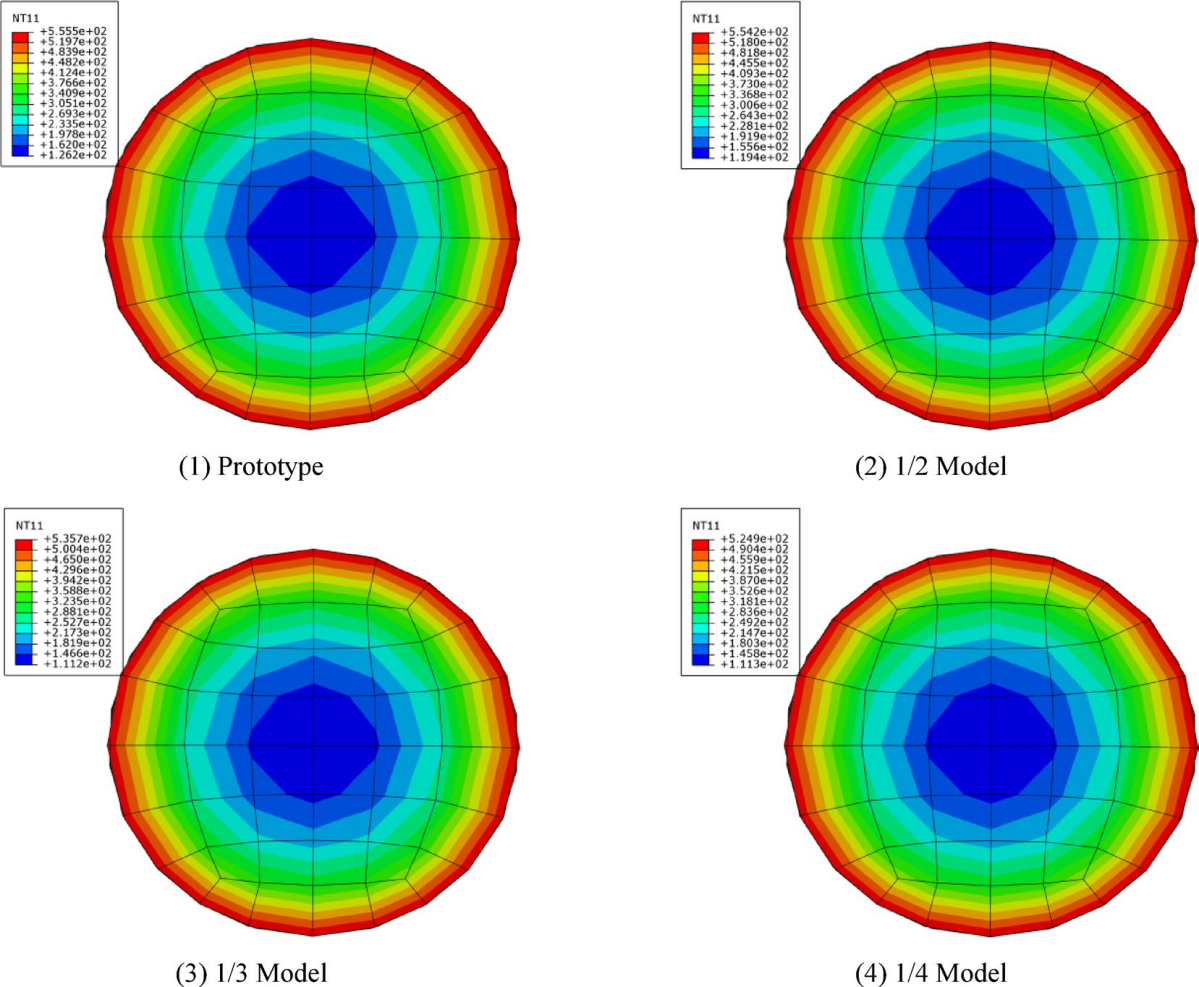
Temperature contour map of the column’s mid-span section.

**Fig. 11:**
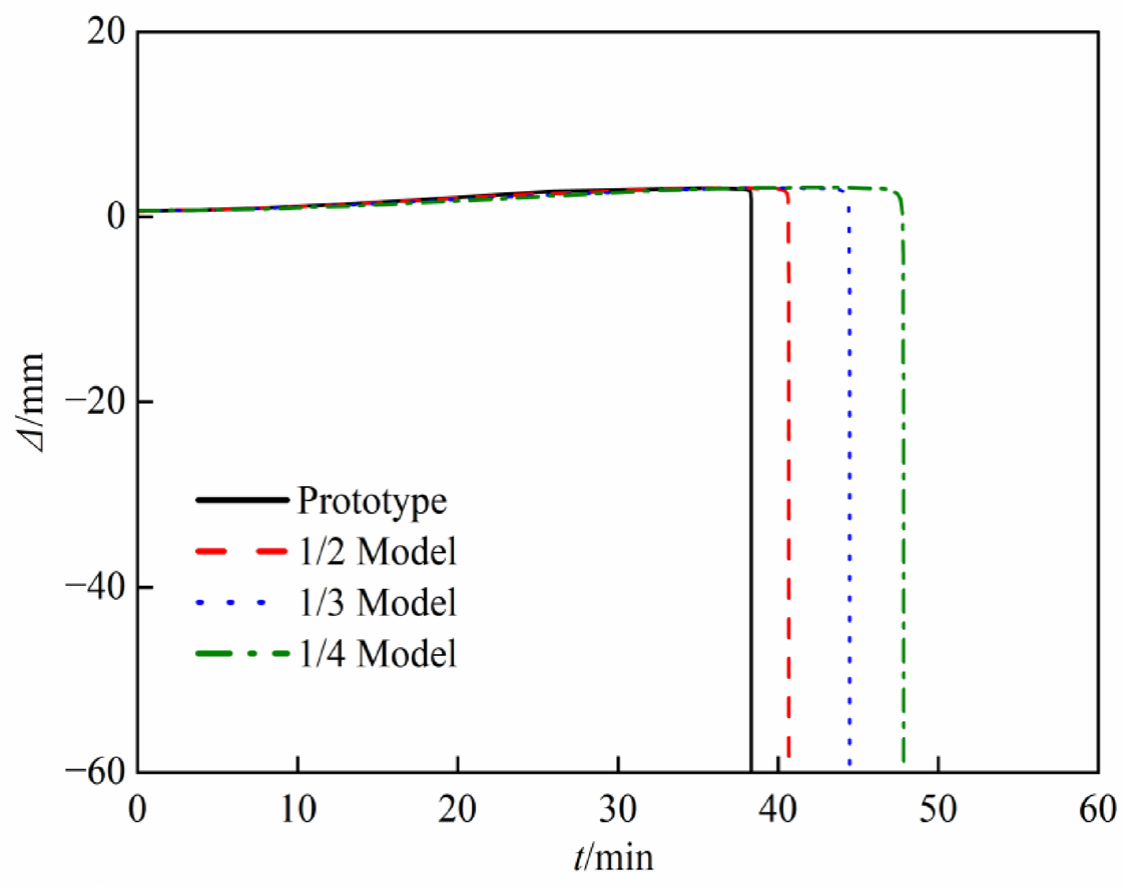
Axial deformation-time (*Δ*-*t*) curves.

The yield strength *f*_y_ of the steel is 360 MPa, and the standard compressive strength of the concrete *f*_cu_ is 45 MPa in the FE model. The axial compressive bearing capacity of the prototype is determined using the formula established by Han^29^:

*N*_u_ = 11558 kN, the thermal parameters are identical to those previously stated, the fire duration time of the prototype is 240 min, and the characteristics and fire duration time of the reduced-scale models are computed based on the similarity relationship presented in Table 5 , where n represents the axial compression ratio. In the fire resistance test, the boundary conditions were set as pinned at both ends, to reduce computational time and allow the member to reach its fire resistance limit more rapidly, a relatively high axial compression ratio of 0.6 was adopted. Figure 10 illustrates the temperature cloud diagram for the mid-span part of the column, while Fig. 11 shows the axial deformation-time curves of the reduced-scale model with different ratios after prototyping.

**Table 5:** Information of similarity models under temperature field similarity theory.

Specimen	*D* × *t*_s_ (mm)	*L* (mm)	n	*e* (mm)	Fire duration (min)
Prototype	432 × 12	7.2	0.6	0	240
1/2 Model	216 × 6	3.6	0.6	0	60
1/3 Model	144	2.4	0.6	0	26.7
1/4 Model	108	1.8	0.6	0	15

Figure 10 illustrates that the temperature color spectrum profiles of both the reduced-scale models and the prototype are congruent, exhibiting no significant temperature gradient discrepancies. The final temperature of the reduced-scale models are slightly lower than that of the prototype, while the final temperature of the 1/4 model is the lowest, exhibiting a maximum temperature difference from the prototype of around 30 °C, which proves that the established temperature heating curves enable the temperature fields of the reduced-scale models and the prototype to maintain a degree of similarity. As shown in Fig. 11, the axial displacement curves of the reduced-scale models’ fire resistance limits, derived after applying time and displacement similarity processing, almost coincide with those of the prototype. However, the fire resistance times of the reduced-scale models consistently exceed that of the prototype, the fire resistance times of the prototype, 1/2, 1/3, and 1/4 reduce-scaled are 38.3 min, 40.7 min, 44.5 min, and 47.8 min, respectively. The reason for this phenomenon is the influence of the size effect on the mechanical properties of concrete at high temperatures, as the size of the specimens increases, the development of deflection also accelerates. In summary, the reduced-scale models of the CFST columns, constructed in accordance with temperature field similarity theory, can reflect the fire resistance performance of the prototype.

## Criterion for lateral impact similarity

### Derivation of similarity criterion

In the lateral impact test of CFST columns, the significant physical quantities include impact duration *t*, dynamic stress *σ*_d_, impact mass *G*, strain rate $\dot{\varepsilon }$, impact velocity *V*, contact force *F,* and displacement *δ*. Let these physical quantities be related by:

18 $$f(t,\sigma_{{\mathrm{d}}} ,G,\dot{\varepsilon },V,F,\delta ) = 0$$

In the aforementioned formula, n = 7, *L*,* T*, and *M* are selected as the fundamental dimensions, so the dimensions of the aforementioned physical quantities are as follows:

$$\left\{ {\begin{array}{*{20}l} {t:T} \hfill \\ {\sigma_{{\mathrm{d}}} :L^{ - 1} T^{ - 2} M} \hfill \\ {G:M} \hfill \\ {\dot{\varepsilon }:T^{ - 1} } \hfill \\ {V:LT^{ - 1} } \hfill \\ {F:LT^{ - 2} M} \hfill \\ {\delta :L} \hfill \\ \end{array} } \right.$$

Selecting velocity *V*, dynamic stress *σ*_d_, and impact mass* G* as the fundamental physical quantities, the dimensional exponent matrix is given as follows:

$$\left| {\begin{array}{*{20}c} 1 & { - 1} & 0 \\ { - 1} & { - 2} & 1 \\ 0 & 0 & 1 \\ \end{array} } \right| = - 3 \ne 0$$

Therefore, the dimensions of the three fundamental physical quantities chosen are mutually independent. According to the *π* theorem, **Eq. **(18) can be expressed as four dimensionless *π* terms.:

19 $$\pi_{1} = \frac{{t^{3} \sigma_{{\mathrm{d}}} V}}{G},\pi_{2} = \frac{{\delta^{3} \sigma_{{\mathrm{d}}} }}{{GV^{2} }},\pi_{3} = \dot{\varepsilon }\left( {\frac{G}{{\sigma_{{\mathrm{d}}} V}}} \right)^{1/3} ,\pi_{4} = \frac{{F^{3} }}{{G^{2} V^{4} \sigma_{{\mathrm{d}}} }}$$

The similarity ratio *β* of each physical quantity related to the reduced-scale model and the prototype can be derived from the *π* term and is defined as:

$$\beta_{{\sigma_{{\mathrm{d}}} }} = \left( {\sigma_{{\mathrm{d}}} } \right)_{{\mathrm{m}}} /\left( {\sigma_{{\mathrm{d}}} } \right)_{{\mathrm{p}}} ,\beta_{\delta } = \delta_{{\mathrm{m}}} /\delta_{{\mathrm{p}}} ,\beta_{{\dot{\varepsilon }}} = \dot{\varepsilon }_{{\mathrm{m}}} /\dot{\varepsilon }_{{\mathrm{p}}} ,\beta_{t} = t_{{\mathrm{m}}} /t_{{\mathrm{p}}} ,\beta_{G} = G_{{\mathrm{m}}} /G_{{\mathrm{p}}} ,\beta_{V} = V_{{\mathrm{m}}} /V_{{\mathrm{p}}} ; \,$$

For *π*_2_, since the displacement of each particle in the reduced-scale model is presumed to be identical to that of the prototype, it follows that *β*_*δ*_ = *β*_*l*_, allowing us to derive:

20 $$\frac{{\delta_{{\mathrm{m}}}^{{3}} \left( {\sigma_{{\mathrm{d}}} } \right)_{{\mathrm{m}}} }}{{G_{{\mathrm{m}}} V_{{\mathrm{m}}}^{2} }} = \frac{{\delta_{{\mathrm{p}}}^{3} \left( {\sigma_{{\mathrm{d}}} } \right)_{{\mathrm{p}}} }}{{G_{{\mathrm{p}}} V_{{\mathrm{p}}}^{2} }} \to \beta_{V} = \sqrt {\frac{{\beta_{l}^{3} \beta_{{\sigma_{{\mathrm{d}}} }} }}{{\beta_{G} }}} \,$$

Given that the density of the impactor is constant, specifically *β*_*ρ*_ = 1, the impact mass *G* can be expressed as *G* = *L*^*3*^*ρ*, *β*_*G*_ = $\beta_{l}^{3}$, leading to the transformation of Eq. (20) into:

21 $$\beta_{{\sigma_{{\mathrm{d}}} }} = \beta_{V}^{2}$$

Correspondingly, the similarity ratios are transformed into expressions containing only *β*_*l*_ and *β*_*V*_, as shown in Table 6.

**Table 6:** Similarity ratio relationship.

Physical quantity	Similarity ratio
Impact velocity *V*	$\beta_{V}^{{}}$
Displacement *δ*	$\beta_{l}$
Strain rate $\dot{\varepsilon }$	$\beta_{V} {/}\beta_{l}$
Dynamic stress *σ*_d_	$\beta_{V}^{2}$
Contact force *F*	$\beta_{V}^{2} \beta_{l}^{2}$
Impact duration *t*	$\beta_{l} {/}\beta_{V}$
Impact mass *G*	$\beta_{l}^{3}$

## Investigation of similarity in high-temperature lateral impact models

### Neglect of material strain rate sensitivity effects

When the material strain rate impact is disregarded, the dynamic stress similarity relationship between the reduced-scale model and the prototype is:

22 $$\left( {\sigma_{{\mathrm{d}}} } \right)_{{\mathrm{m}}} {/}\left( {\sigma_{{\mathrm{d}}} } \right)_{{\mathrm{p}}} = \beta_{V}^{2} = 1,\beta_{V}^{{}} = 1 \,$$

Based on the finite element modeling methodology and parameter configurations outlined in Chapter 1, the similarity criterion obtained from Table 6, adopting the model dimensions from Table 5, the prototype, along with its 1/2, 1/3, and 1/4 reduced-scale models were established. When determining the mesh size for the prototype, it was observed that varying the mesh size within the range of 40 mm to 80 mm has a negligible influence on the simulation results, with the primary difference being reflected in the computational time. However, when the mesh size is reduced to below 40 mm, the analysis fails to converge; when it exceeds 80 mm, severe hourglassing occurs. So the finite element model of the prototype has a mesh size of 60mm, while the mesh size of the reduced-scale models are modified in accordance with *β*_*l*_. Table 7 displays the parameters of each model, where *G* represents the impact mass, *V* denotes the impact velocity, and n indicates the axial compression ratio. Since the influence of the internal temperature gradient of concrete can be neglected when the strain rate is below the transition strain rate, the temperature of the steel tube outer surface, *T*, is taken as the temperature of the structural member, to analyze the similarity of the lateral impact reaction between the reduced-scale models and the prototype at varying temperatures of *T* = 100 °C, *T* = 300 °C, and *T* = 500 °C.

**Table 7:** Information of similarity models for lateral impact under high temperature.

Specimen	*G* (kg)	*V* (m/s)	*D* × *t*_s_ (mm)	*L* (m)	n	Fire duration (min)		
						*T* = 100 °C	*T* = 300 °C	*T* = 500 °C
Prototype	6480	6	432 × 12	7.2	0.3	35.7	101	203.3
1/2 Model	810	6	216 × 6	3.6	0.3	8.9	25.3	50.8
1/3 Model	270	6	144 × 4	2.4	0.3	3.97	11.2	22.6
1/4 Model	101.25	6	108 × 3	1.8	0.3	2.23	6.3	12.7

**Fig. 12:**
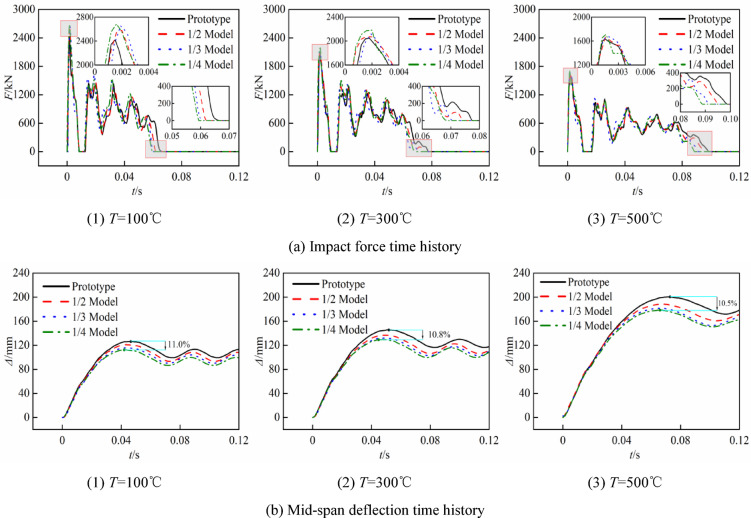
Time curve of prototype and similar model.

Figure 12 illustrates the comparison of the lateral impact time history curves for each group of reduced-scale and prototype at high temperatures, while Table 8 documents the simulation findings and associated errors, where* F*_p_ is the platform value of the impact force curve,* Δ*_max_ is the maximum deflection at the mid-span, *t* is the impact time, and *η* is the error associated with each numerical value of both the reduced-scale model and the prototype.

**Table 8:** Simulation results and errors between scaled-down models and prototypes.

Temperature	Specimen	*F*_p_ (kN)	*η*_*F*_ (%)	*Δ*_max_ (mm)	*η*_*Δ*_ (%)	*t* (ms)	*η*_*t*_ (%)
100℃	Prototype	838.45		126.28		66.7	
	1/2 Model	882.26	5.2	121.00	4.2	62.0	7.0
	1/3 Model	897.49	7.0	115.86	8.3	60.5	10.2
	1/4 Model	920.35	9.8	112.41	11.0	58.4	12.4
300℃	Prototype	782.31		145.60		77.4	
	1/2 Model	819.35	4.7	136.50	6.3	73.4	5.2
	1/3 Model	832.65	6.4	132.08	9.3	70.1	9.4
	1/4 Model	845.51	8.1	129.81	10.8	68.2	11.9
500℃	Prototype	581.68		200.40		98.7	
	1/2 Model	590.72	1.6	188.40	6.0	95.2	3.5
	1/3 Model	596.46	2.5	181.54	9.4	91.2	7.6
	1/4 Model	608.57	4.6	179.41	10.5	88.8	10.0

Figure 12 and Table 8 illustrate the following:

After restoring the time-history curves of the reduced-scale model according to the similarity ratio, the time-history curves of the reduced-scale models show that, the trends of the impact force, deflection, and impact duration curves are essentially identical to those of the prototype at the same temperature. Due to the neglect of strain rate sensitivity, there are some discrepancies between the reduced-scale models and those of the prototype, the mechanical properties of the reduced-scale models are slightly higher than those of the prototype, and as the scaling ratio decreases, all errors progressively increase. The maximum errors in the impact force platform value, mid-span deflection, and impact time between the reduced-scale model and the prototype are 8.3%, 11.0%, and 12.4%, respectively, derived from the 1/4 model at 100 °C.

Comparing the impact curves and mid-span deflection curves of the prototypes at 100 °C, 300 °C, and 500 °C reveals that the impact resistance of the CFST columns diminishes with rising temperature, this is evidenced by the reduction in the impact force platform values (838.45 kN, 782.31 kN, 581.68 kN), the increase in mid-span deflection (126.28 mm, 145.60 mm, 200.40 mm), the extension of impact duration (0.067 s, 0.077 s, 0.099 s), and the prolongation of the impact force zero value time. Qu et al.^40^ indicates that in the impact testing of CFST columns, the core concrete typically contributes only 20% to 30% to the flexural bearing capacity of the components. The high-temperature constitutive relationship of steel indicates that its mechanical properties markedly diminish at 500 °C, resulting in significant discrepancies in the impact force platform value, impact duration, and deflection of the 500 °C prototype compared to the 100 °C and 300 °C models, which exhibit minimal differences between each other.

When comparing the errors of the impact force platform values of models with identical scale ratios at varying temperatures (for instance, the errors for the 1/2 model at 100 °C, 300 °C, and 500 °C are 5.2%, 4.7%, and 1.6%, respectively, corresponding to reductions of 9.62% and 65.96% in sequence.), it is observed that the error in the impact force platform values diminishes as temperature increases. This phenomenon can be attributed to two primary factors: firstly, as the duration of heating increases, the discrepancy between the fitted heating curve and the original data decreases, leading to a reduction in the error between the temperature fields of the reduced-scale models and the prototype, thereby aligning the mechanical properties of the materials more closely; secondly, at low strain rates, the detrimental effects of high temperatures on the mechanical properties of the material outweigh the enhancements induced by the strain rate effect, thereby mitigating the distortion associated with the strain rate effect.

### Consideration of material strain rate sensitivity effects

The preceding analysis revealed that, when the influence of material strain rate sensitivity is neglected, discrepancies exist between the reduced-scale models and the prototype, the mechanical properties of all the reduced-scale models are higher than those of the prototype, resulting in an unsafe prediction of prototype behavior, therefore, the reduced-scale models require correction. In the present study, Drazetic et al.^41^ and Oshiro et al.^42^ employed a method known as non-direct similarity to modify the impact velocity of the reduced-scale model by utilizing the exponential constitutive relation to derive the factor *β*_*V*_ associated with the impact velocity, thereby mitigating the distortion induced by the strain rate effect.

For the concrete, the exponential constitutive relationship shown in Eq. (5) is selected for the derivation, as follows:

23 $$\begin{aligned} & \beta_{{\sigma_{{\mathrm{d}}} }} = \frac{{\left( {\sigma_{{\mathrm{d}}} } \right)_{{\mathrm{m}}} }}{{\left( {\sigma_{{\mathrm{d}}} } \right)_{{\mathrm{p}}} }} = \frac{{\sigma_{0} \left( {\dot{\varepsilon }_{{\mathrm{m}}} /\dot{\varepsilon }_{0} } \right)^{{1.026\alpha_{{\mathrm{s}}} }} }}{{\sigma_{0} \left( {\dot{\varepsilon }_{{\mathrm{p}}} /\dot{\varepsilon }_{0} } \right)^{{1.026\alpha_{{\mathrm{s}}} }} }} = \left( {\frac{{\dot{\varepsilon }_{{\mathrm{m}}} }}{{\dot{\varepsilon }_{{\mathrm{p}}} }}} \right)^{{1.026\alpha_{{\mathrm{s}}} }} = \left( {\beta_{{\dot{\varepsilon }}} } \right)^{{1.026\alpha_{{\mathrm{s}}} }} = \left( {\frac{{\beta_{V} }}{{\beta_{l} }}} \right)^{{1.026\alpha_{{\mathrm{s}}} }} \\ & \quad \quad \beta_{V} { = }\beta_{l}^{{\frac{{1.026\alpha_{{\mathrm{s}}} }}{{1.026\alpha_{{\mathrm{s}}} - 2}}}} \\ \end{aligned}$$

For steel, the constitutive relationship given in Eq. (2) is selected for the derivation, as follows:

24 $$\beta_{{\sigma_{{\mathrm{d}}} }} = \frac{{\left( {\sigma_{{\mathrm{d}}} } \right)_{{\mathrm{m}}} }}{{\left( {\sigma_{{\mathrm{d}}} } \right)_{{\mathrm{p}}} }} = \frac{{\left( {\sigma_{{\mathrm{s}}} } \right)_{{\mathrm{m}}} \left[ {1 + \left( {\frac{{\dot{\varepsilon }_{{\mathrm{m}}} }}{{C_{{\mathrm{m}}} }}} \right)^{{1/p_{{\mathrm{m}}} }} } \right]}}{{\left( {\sigma_{{\mathrm{s}}} } \right)_{{\mathrm{p}}} \left[ {1 + \left( {\frac{{\dot{\varepsilon }_{{\mathrm{p}}} }}{{C_{{\mathrm{p}}} }}} \right)^{{1/p_{{\mathrm{p}}} }} } \right]}} = \beta_{{\sigma_{{\mathrm{s}}} }} \cdot \frac{{1 + \left( {\frac{{\beta_{V} \dot{\varepsilon }_{{\mathrm{p}}} }}{{\beta_{l} C_{{\mathrm{m}}} }}} \right)^{{1/p_{{\mathrm{m}}} }} }}{{1 + \left( {\frac{{\dot{\varepsilon }_{{\mathrm{p}}} }}{{C_{{\mathrm{p}}} }}} \right)^{{1/p_{{\mathrm{p}}} }} }} \,$$

Since the reduced-scale models and the prototype employ the same materials,$\beta_{{\sigma_{{\mathrm{s}}} }} { = }1,C_{{\mathrm{m}}} { = }C_{{\mathrm{p}}} { = }400,p_{{\mathrm{m}}} { = }p_{{\mathrm{p}}} { = }1$, by substituting the similarity relation $\beta_{{\sigma_{{\mathrm{d}}} }} { = }\beta_{V}^{2}$ into Eq. (24) , we obtain:

25 $$\beta_{V}^{2} { = }\frac{{400{ + }\frac{{\beta_{V} }}{{\beta_{l} }}\dot{\varepsilon }_{{\mathrm{p}}} }}{{400 + \dot{\varepsilon }_{{\mathrm{p}}} }}$$

**Fig. 13:**
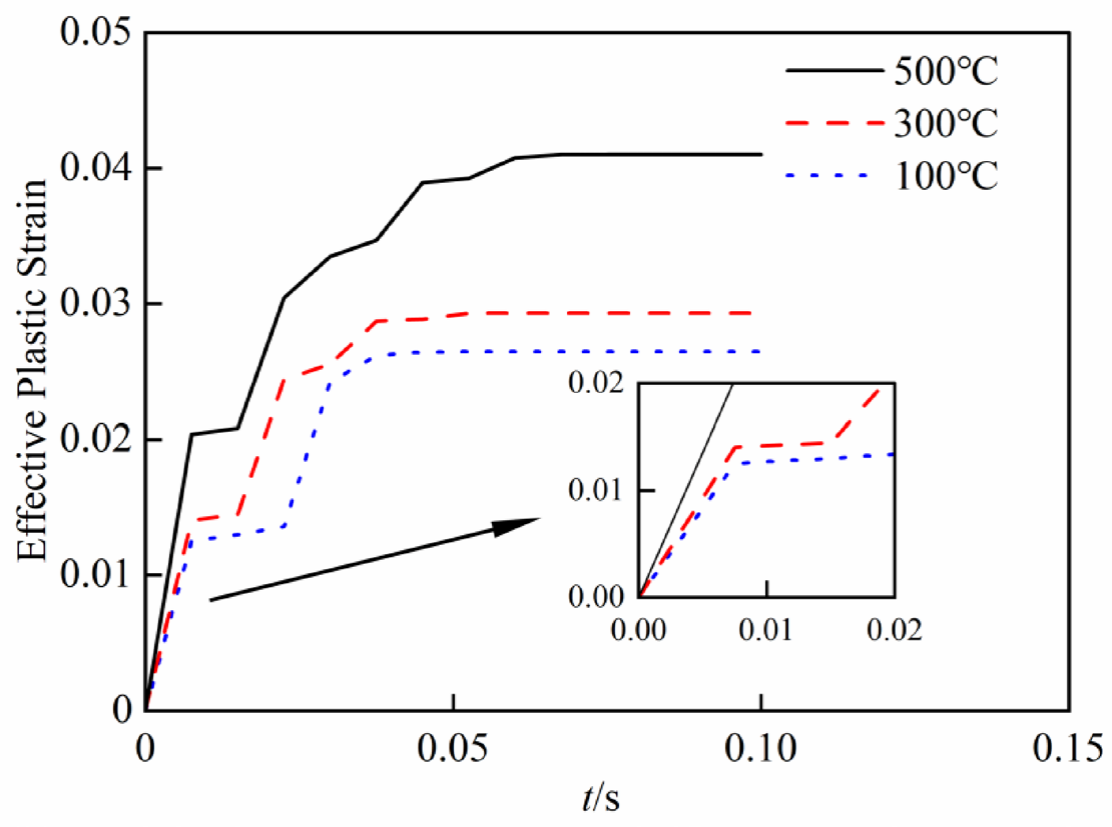
Equivalent plastic strain–time curve of the prototype.

**Fig. 14:**
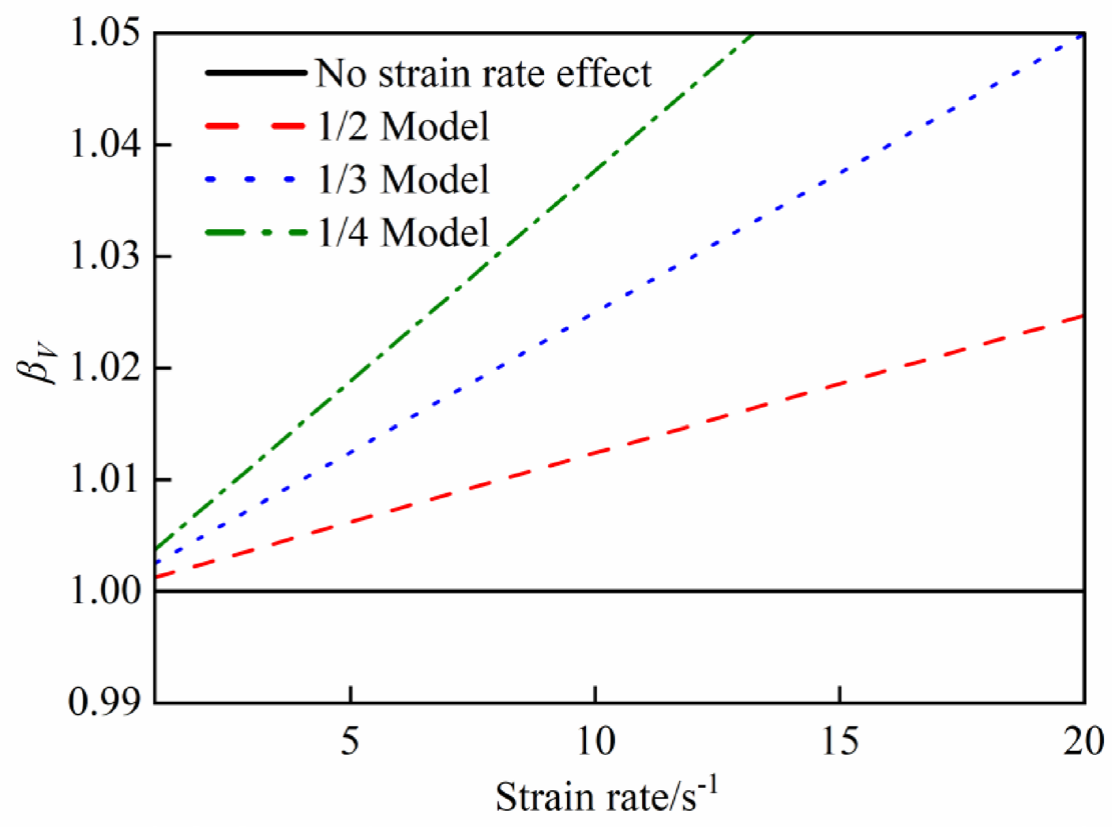
*β*_*V*_ (Dynamic behavior of steel).

Equation (25) delineates the functional link between the velocity similarity ratio *β*_*V*_ and the strain rate $\dot{\varepsilon }_{{\mathrm{p}}}$ of the prototype, $\dot{\varepsilon }_{{\mathrm{p}}}$ can be derived from the strain–time curve of the prototype. Figure 13 illustrates the effective plastic strain–time curve of the prototype at various temperatures. The maximum strain rates at each temperature are selected to correct the velocity similarity ratio. The corresponding maximum strain rates at 100 °C, 300 °C, and 500 °C are 1.67, 1.87, and 2.71, respectively. The *β*_*V*_-$\dot{\varepsilon }_{{\mathrm{p}}}$ curves under different *β*_*l*_ are shown in Fig. 14.

The velocity similarity ratio *β*_*V*_ derived from Eqs. (23) and (25) is presented in Table 9 .

**Table 9:** Similar ratios of impact velocities calculated by different strain rate constitutive equations (*β*_*V*_).

Temperature	*β*_*l*_	*β*_*V*1_ (Concrete)	*β*_*V*2_ (Steel)
100 °C	1/2	1.008	1.0021
	1/3	1.013	1.0042
	1/4	1.016	1.0063
300 °C	1/2	1.008	1.0023
	1/3	1.013	1.0047
	1/4	1.016	1.0070
500 °C	1/2	1.008	1.0034
	1/3	1.013	1.0068
	1/4	1.016	1.0102

**Fig. 15:**
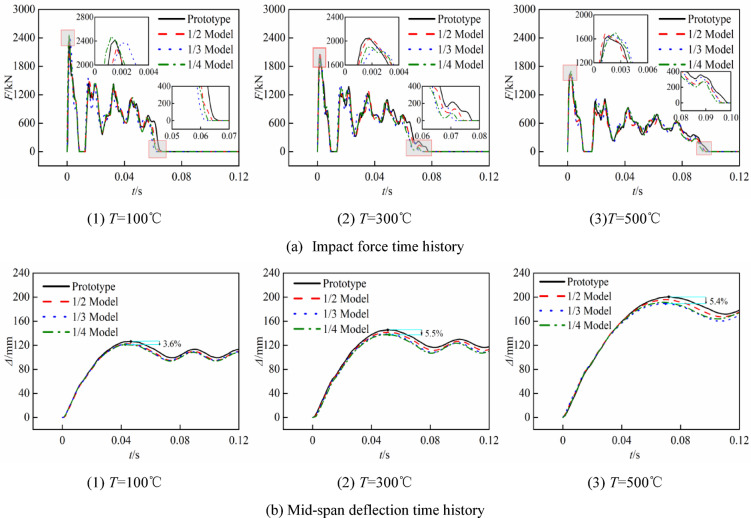
Time curve of prototype and similar model after correction (*β*_*V*1_).

**Fig. 16:**
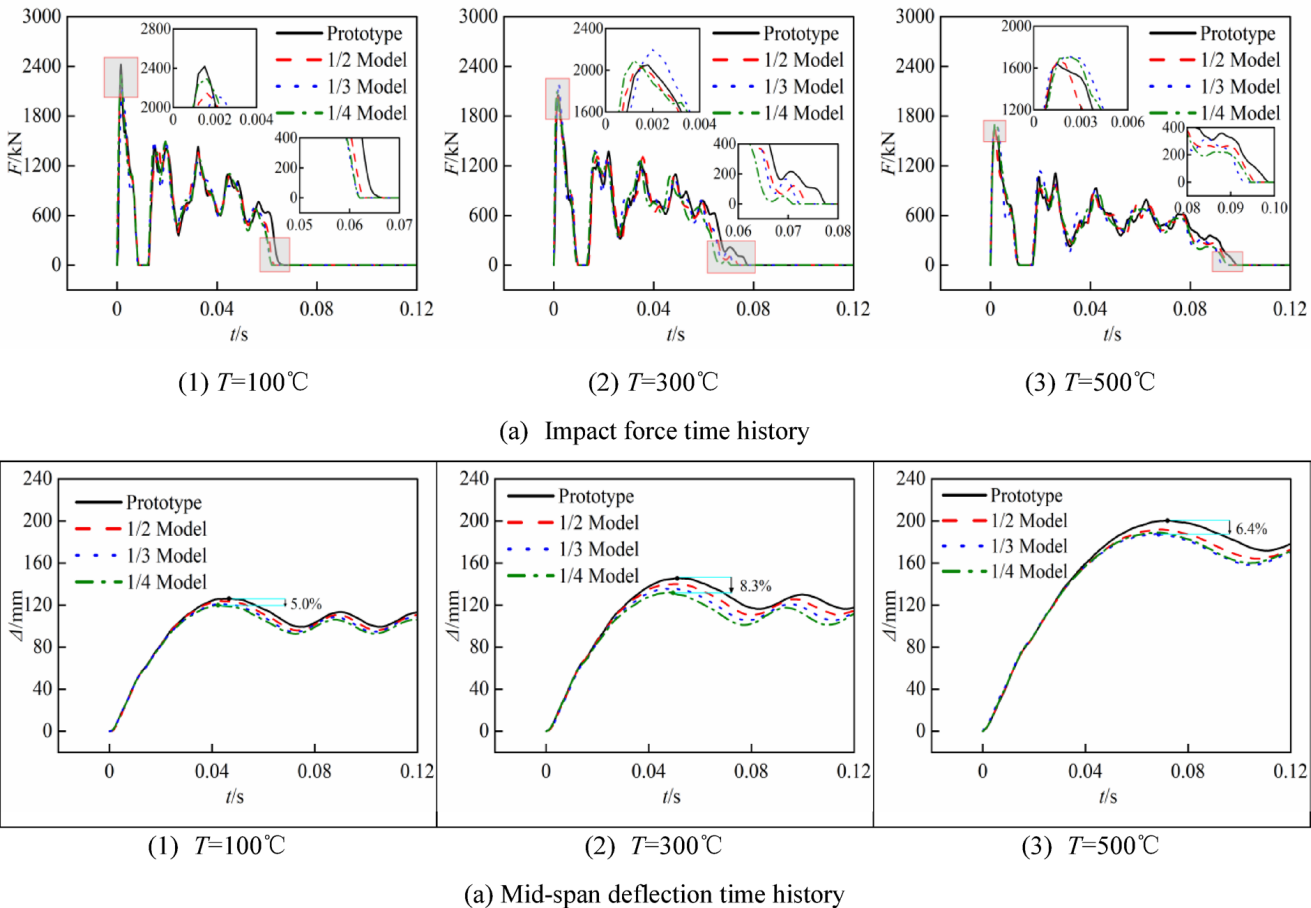
Time curve of prototype and similar model after correction (*β*_*V*2_).

The reduced-scale models are corrected using the velocity similarity ratio *β*_*V*_, derived from the concrete and steel constitutive relationships, a comparison of the lateral impact time-history curves between each set of reduced-scale models and the prototype at high temperatures is shown in Figs. 15 and 16. Tables 10 and 11 document the results and errors after the corrections using *β*_*V*1_ and *β*_*V*2_, respectively.

**Table 10:** Simulation results and errors between scaled-down models and prototypes after correction (*β*_*V*1_).

Temperature	Specimen	*F*_p_ (kN)	*η*_*F*_ (%)	*Δ*_max_ (mm)	*η*_*Δ*_ (%)	*t* (ms)	*η*_*t*_ (%)
100 °C	Prototype	838.45		126.28		66.7	
	1/2 Model	857.46	2.3	122.62	2.9	64.1	3.9
	1/3 Model	832.45	0.7	121.83	3.5	61.4	7.9
	1/4 Model	871.51	3.9	121.74	3.6	62.6	6.1
300 °C	Prototype	782.31		145.60		77.4	
	1/2 Model	793.99	1.5	141.55	2.8	74.6	3.6
	1/3 Model	789.34	0.9	138.81	4.7	71.1	8.1
	1/4 Model	784.49	0.3	137.63	5.5	72.7	6.1
500 °C	Prototype	581.68		200.40		98.7	
	1/2 Model	574.72	1.2	195.99	2.2	96.4	2.3
	1/3 Model	570.71	1.9	189.51	5.4	95.1	3.6
	1/4 Model	591.17	1.6	191.58	4.4	94.2	4.6

**Table 11:** Simulation results and errors between scaled-down models and prototypes after correction (*β*_*V*2_).

Temperature	Specimen	*F*_p_ (kN)	*η*_*F*_ (%)	*Δ*_max_ (mm)	*η*_*Δ*_ (%)	*t* (ms)	*η*_*t*_ (%)
100 °C	Prototype	838.45		126.28		66.7	
	1/2 Model	871.15	3.9	123.94	1.9	63.2	5.2
	1/3 Model	839.91	0.2	120.90	4.3	61.9	7.2
	1/4 Model	854.23	1.9	119.91	5.0	61.9	7.2
300 °C	Prototype	782.31		145.60		77.4	
	1/2 Model	791.81	1.2	139.10	4.5	73.8	4.7
	1/3 Model	794.93	1.6	135.54	6.9	72.3	6.6
	1/4 Model	809.52	3.5	133.47	8.3	70.9	8.4
500 °C	Prototype	581.68		200.40		98.7	
	1/2 Model	577.24	0.8	192.03	4.2	95.5	3.2
	1/3 Model	577.73	0.7	187.60	6.4	93.2	5.6
	1/4 Model	579.09	0.4	188.90	5.7	94.6	4.2

The comparative study indicates that once the reduced-scale model is adjusted using the velocity similarity factor obtained from the strain rate constitutive model of concrete and steel, the time history curve of the reduced-scale model aligns more closely with that of the prototype. Tables 10 and 11 illustrate a further reduction in the mapping error of the reduced-scale model to the prototype. This approach exhibits the most effective correction for the impact force platform value, with a corrected error range of 3.9% to 0.02%; the corrected error for mid-span deflection ranges from 8.3 to 1.9%; and the error for impact time ranges from 8.4 to 2.3%. Using the 1/4 reduced-scale model with the highest error at 100 °C as a reference, the error in the impact force platform value decreases from 8.3 to 3.9% and 1.9% following corrections by *β*_*V*1_ and *β*_*V*2_; the error in mid-span deflection diminishes from 11.0 to 3.6% and 5.0%; and the error in impact time is reduced from 12.4 to 6.1% and 7.3%.

The mean errors of the impact force platform value, mid-span deflection, and impact time, following correction by *β*_*V*1_ and *β*_*V*2_, are 1.58%, 3.89%, 5.13% and 1.58%, 5.24%, 5.81%, respectively. At low strain rates, the correction effects of the two constitutive relations on the impact force platform value are comparable; however, the correction effect of *β*_*V*1_ on mid-span deflection and impact duration is markedly superior to that of *β*_*V*2_.

## Conclusion

This study investigates the similarity of lateral impact responses in CFST columns of different scales under the coupled effects of high temperature and axial load, when the strain rate is below the transition strain rate. Initially, based on the temperature field similarity criterion, the 1/4 scale model was heated following the ISO-834 standard heating curve, and temperature data were collected. From these data, the corresponding temperature curves for the prototype and other reduced-scale models were derived, ensuring consistency in the temperature fields between the reduced-scale models and the prototype throughout the heating process. Subsequently, based on the similarity criterion for lateral impact, the relationship of the similarity ratio for each physical quantity in the lateral impact model was calculated utilizing the *V*–σ_d_–*G* dimension, and the similarity between the reduced-scale model and the prototype at three distinct temperatures was investigated. Finally, the velocity similarity ratio *β*_*V*_ was derived separately from the strain-rate-sensitive constitutive equations for concrete and steel, and corrections were implemented to address the distortion effects caused by strain rate. The following conclusions are drawn:

1. Based on the temperature field similarity criterion, to ensure consistency in the temperature fields between the reduced-scale model and the prototype, the heat flux density for the reduced-scale model must be higher than that for the prototype, and after time scaling, the heating rate of the furnace temperature in the reduced-scale model becomes significantly faster than that of the prototype. The smallest scale model was heated in accordance with the ISO-834 standard heating curve, and the temperature data on the outer surface of its steel tube was collected. Using a thermal radiation model, the heating curves for the prototype and other larger-scale models were derived. As a result, the temperature fields obtained for the various reduced-scale models demonstrated good similarity with the prototype.
2. When the influence of strain rate effects is not considered, the reduced-scale model for lateral impact on circular CFST columns at high temperatures, derived based on similarity theory, can predict the mechanical properties of the prototype reasonably well to a certain extent. Nevertheless, the prediction is unreliable due to the superior mechanical properties of the reduced-scale model compared to the prototype. As the scaling ratio diminishes, the discrepancy between the reduced-scale model and the prototype will progressively escalate, as the temperature rises, the discrepancy between the reduced-scale model and the prototype will progressively diminish, this is because the degradation of material mechanical properties at high temperatures reduces the distortion caused by strain rate effects.
3. To reduce the impact of strain rate effects on the mechanical properties of CFST columns subjected to lateral impact at high temperatures, the velocity similarity ratio *β*_*V*_ was formulated to adjust the reduced-scale model in accordance with the strain rate constitutive equations of concrete and steel. Following the comparison and analysis of finite elements, the discrepancies in contact force, maximum deflection, impact force platform value, and time history curve between the reduced-scale model and the prototype were significantly minimized.
4. The lack of coupled fire-impact tests imposes certain limitations on the validation of the proposed model. The finite element model established in this paper adopts the assumption of ideal geometric scalability, however, in practical applications, it is not convenient to fabricate scaled models in which all dimensions satisfy the same geometric similarity ratio. The simulation in this paper focuses on the impact process at low strain rates and does not involve specimen fracture, the similarity of material fracture under high strain rates requires further investigation.

## Data Availability

The data and Abaqus files used in this study are available upon reasonable request from the corresponding author.
